# Merging neural stimulation and exoskeletons to enhance sensorimotor hand functions after brain or spinal cord injury

**DOI:** 10.1126/sciadv.ady3144

**Published:** 2026-06-19

**Authors:** Andrea Cimolato, Dunja Cekić, Natalija Katić Šećerović, Margerita Razzoli, Francesco Missiroli, Jan Dittli, Pietro Palopoli, Sara Bellomo, Julia Kenel, Suzana Dedijer Dujović, Sebastian Frese, Lorenzo Masia, Olivera Djordjevic, Olivier Lambercy, Giacomo Valle, Ljubica Konstantinovic, Stanisa Raspopovic

**Affiliations:** ^1^ Center for Medical Physics and Biomedical Engineering, Medical University of Vienna, Währinger Gürtel 18-20, 1090 Vienna, Austria.; ^2^ Comprehensive Center for Artificial Intelligence in Medicine, Medical University of Vienna, Währinger Gürtel 18-20, 1090 Vienna, Austria.; ^3^ NeuroEngineering Laboratory, Department of Health Sciences and Technology, ETH Zurich, Zurich, Switzerland.; ^4^ Institute Mihajlo Pupin, University of Belgrade, Belgrade, Serbia.; ^5^ Rehabilitation Engineering Laboratory, Department of Health Sciences and Technology, ETH Zurich, Zurich, Switzerland.; ^6^ Munich Institute of Robotics and Machine Intelligence, School of Computation Information and Technology, Technical University of Munich, Munich, Germany.; ^7^ Vilje Bionics AS, Hausmanns gate 16, 0182 Oslo, Norway.; ^8^ Clinic for Rehabilitation “Dr Miroslav Zotović,” Belgrade, Serbia.; ^9^ Technology and Innovation Unit and Department of Research, ZURZACH Care AG, Bad Zurzach, Aargau, Switzerland.; ^10^ Faculty of Medicine, University of Belgrade, Belgrade, Serbia.; ^11^ Future Health Technologies, Singapore-ETH Centre, Campus for Research Excellence and Technological Enterprise, Singapore, Singapore.

## Abstract

Sophisticated hand movements are essential for daily activities, but central neurological impairments often compromise them. Since full recovery through conventional physiotherapy is rare, assistance is crucial. While neural implants show promise, clinical use remains distant, urging immediate assistive alternatives. Current exoskeletons and neurostimulation garments lack sufficient motor support and sensory feedback, limiting dexterity. We developed a neurorobotic system combining portable exoskeletons with targeted neurostimulation via custom-made e-sleeve and tested it in 14 individuals with central neural injuries. We provide evidence of restored finger mobility and tactile perception, even in patients with clinically complete sensory loss, by recruiting residual peripheral pathways. Eight participants completed functional assessments, in which they exploited neurostimulation to improve grasp precision and enhance strength. This enabled manipulation of fragile and cumbersome objects, essential for everyday activities. Personalized assistive technologies have clinical potential to promote independence and support the reintegration of people with neurological impairments into society.

## Teaser

SensoExo restores hand sensation and everyday object grasping in neurological patients combining robotics and neurostimulation.

## INTRODUCTION

Hand motor and sensory impairments, resulting from neurological disorders or traumatic injuries, pose notable challenges for more than 50 million individuals worldwide (*1*, *2*). These impairments can stem from a diverse cohort of conditions, such as stroke (*3*–*5*), spinal cord injury (SCI) (*6*), or traumatic brain injury (*7*, *8*). It is estimated that around 70% of stroke (*9*) survivors and 55% of patients with SCI (*10*) are affected by lasting hand impairments. These impairments affect their ability to perform basic activities of daily living (ADL) and interact effectively with the environment (*11*–*14*) (e.g., dressing, feeding, and personal hygiene). For these reasons, individuals with hand impairments may also experience difficulties in social integration and acceptance (*15*–*17*). The limitations imposed by their condition may lead to feelings of frustration, isolation, and depression (*17*, *18*). Physical and occupational physiotherapy [including exercises like object grasp-and-release task (GRT), intensive bimanual arm functional training, and constraint-induced therapy] continues to be the primary treatment approach for these patients (*19*). However, patients often reach a recovery plateau, or the treatment provides only short-term effects without retention, leaving individuals with limited residual abilities and relying on compensatory strategies (*20*–*23*). Therefore, there is an identified need for developing daily assistive devices that would support patients in fulfilling their ADL (*24*–*26*) and enhance their functional performance (*27*). Robotic assistive devices, such as exoskeletons, show promise in addressing sensorimotor impairments (*28*), through integrated actuation systems that support the flexion and extension of the fingers. Portable assistive exoskeletons are gaining interest for their assistance in different grasp types and grip force while being compact, lightweight, and more compliant with environment interactions compared to standard rehabilitative rigid exoskeletons (*29*–*31*). By exploiting properties of soft materials, such as elastomers and flexible cables (*32*), these devices offer multiple advantages, such as extended wearability without causing patient fatigue, providing greater comfort and mobility for ADL (*19*, *33*–*35*). However, the hand has 20 degrees of freedom (DoF), all of which are involved for high control precision to perform common daily tasks. These DoF are usually only partially restored in assistive exoskeletons, as every additional actuation or transmission system adds to the weight and complexity of the system (*32*). For this reason, portable assistive devices struggle to generate sufficient force for desired movements, posing challenges for individuals with no residual movement or spasticity to effectively use the exoskeleton (*36*, *37*). On the other hand, while many exoskeletons leave the palms and fingertips free to allow for natural somatosensory interaction, there is no solution for users who suffer from damage or loss of sensory feedback in the hand. No portable assistive hand exoskeleton has integrated artificial sensory feedback, providing users with functionally relevant sensory information (*38*).

In the pursuit of lightweight and adaptable assistive devices, wearable electrical stimulation garments have emerged as a promising avenue for motor assistance in hand functions (*39*, *40*). These devices leverage the natural actuators (muscles and nerves) of the human body, eliminating the need for additional mechanical power (*41*). Specifically, e-sleeves are designed to deliver controlled electrical currents through embedded electrodes, stimulating the nerves and muscles in the forearm (*42*–*48*). While current research is focused on developing these e-sleeves, there are now no commercially available solutions for patients (*49*). E-sleeves are primarily designed with the intended purpose of creating an interface with the muscles, allowing for the reading of muscles’ electromyography for intention detection and for the application of functional electrical stimulation (FES) to generate movement (*49*). However, e-sleeves have a limited ability to provide precise and stable movement control due to challenges in achieving selective stimulation at the single muscle motor point (*50*). Difficulties arise from transcutaneous identification of the electrode location and the presence of deep muscles, making it challenging to accurately place the electrodes on the skin for targeted stimulation. Limited evidence is provided regarding the restoration of sensory feedback for patients with hand sensory loss, despite the growing interest in transcutaneous electrical stimulation (*51*–*57*). Last, there is now no available solution using e-sleeves for both sensory and motor assistance.

Invasive alternatives to e-sleeve that deliver electrical stimulation directly to muscles and nerves have demonstrated some potential in selectively recruiting specific muscle groups. However, most of the supporting evidence derives from animal studies (*58*–*60*), particularly in nonhuman primates, with human research largely limited to preliminary proof-of-concept trials (*61*, *62*). Consequently, the benefits of such invasive procedures do not currently outweigh the risks associated with multiple implant surgeries, especially in typically frail populations.

The variability in hand disabilities poses a notable challenge in developing assistive devices able to effectively accommodate diverse severity of motor and sensory impairments (*56*, *63*). Existing assistive devices often struggle to provide the required adaptability in ADL (*64*, *65*) depending on user disability range, as for example the level of SCI or stroke severity (*3*, *66*, *67*). To add complexity to the situation, conventional clinical assessment and scales often fall short in capturing the extensive range of disabilities, resulting in different unmet needs even among individuals classified under the same category (*68*–*70*).

To tackle these limitations of robotic assistive technologies, in this work, we developed a SensoExo device, composed of two main parts: (i) a custom-made transcutaneous electrical stimulating garment, NeuroSleeve, designed through data-driven optimization of electrode size and placement to enable selective finger somatotopic sensory and motion restoration, combined with (ii) an assistive, customized portable exoskeleton. NeuroSleeve restores somatotopic tactile sensation in the impaired hand via a transcutaneous electrical nerve stimulation (TENS) module, modulated via wearable fingers pressure sensors. Furthermore, NeuroSleeve offers grasp assistance through a FES module, situated on the proximal section of the forearm. It effectively stimulates the flexor digitorum (FD) and extensor digitorum (ED) muscles, facilitating hand opening and closing movements, while increasing the gripping force. Two different types of exoskeletons were constructed and used symbiotically with NeuroSleeve to demonstrate the garment’s adaptability and superior assistance regardless of the specific device: modified RELab tenoexo (RELab, ETH Zurich, Switzerland) (*19*, *71*, *72*) and a specifically developed CATCHGlove (ARIES Lab, Heidelberg University, Germany). These both share control with NeuroSleeve and provide a custom-made modular hand module. SensoExo was tested on 14 participants affected by stroke, SCI, or brain injury.

While several individual components of the system have been previously explored (*29*, *35*, *36*, *39*, *44*), the integration of somatotopic sensory restoration via TENS (*73*–*77*) within a wearable grasping assistive device represents the primary novelty of this work. Importantly, our results provide unique evidence that noninvasive TENS can restore functionally meaningful tactile feedback even in individuals with complete clinical sensory loss due to central nervous system damage, by selectively recruiting afferent pathways that are not effectively recruited during unassisted grasping. With promising potential, NeuroSleeve serves as an adaptive technology, customizing and enhancing assistance from portable assistive hand exoskeletons to meet patients’ specific needs in everyday activities, highlighting the importance of personalized assistive and rehabilitative technologies.

## RESULTS

### Combining assistive exoskeleton with superficial electrical neurostimulation

**Fig. 1.**
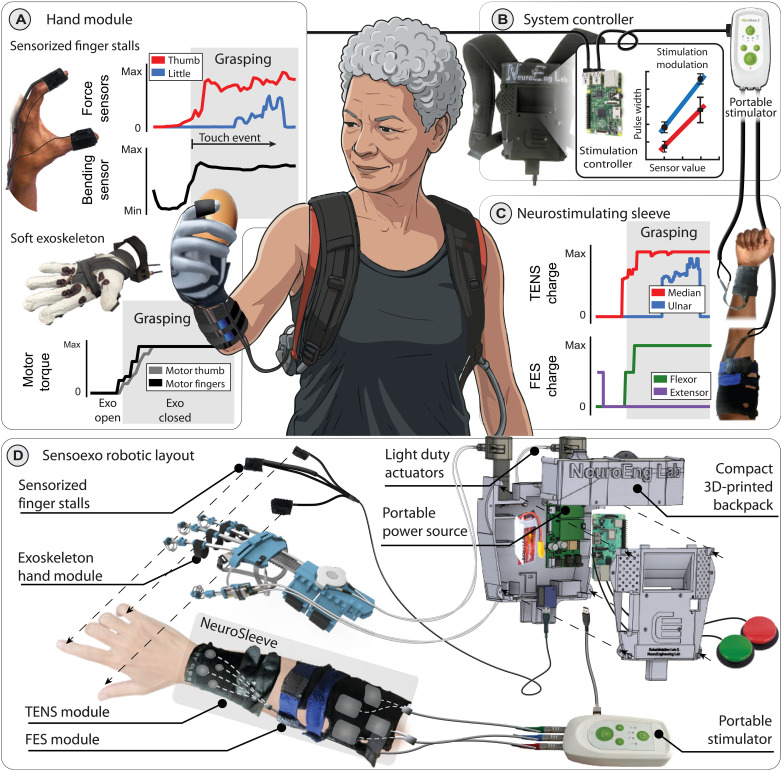
SensoExo system and human-in-the-loop control. (**A**) The hand module consists of two main parts: the sensorized finger stalls with embedded force sensors and a bending sensor on the index finger, serving as a sensing unit to measure multiple contact forces during grasping and finger movements; the portable assistive hand exoskeleton. (**B**) The stimulation controller, using an embedded microcontroller, linearly maps the stimulation pulse width based on the recorded force sensor values. It enables real-time delivery of both FES and TENS. (**C**) The NeuroSleeve TENS and FES modules are wired and connected to the stimulator, delivering current to the median and ulnar nerves and the finger flexor and extensor muscles, respectively, through embedded conductive textile electrodes. The stimulation is synchronized with the exoskeleton control, allowing the user to simultaneously benefit from the assistance of both systems. (**D**) SensoExo robotics components, including a developed hand module with flex-sensor control for intention detection in patients with residual mobility (or buttons used when lacking hand mobility), sensorized finger stalls recording grasp force from the hand exoskeleton, and a NeuroSleeve for neurostimulation. All components connect to a battery-powered, 3D-printed backpack housing the motors and electronic components.

We developed SensoExo as the combination of a personalized neurostimulation garment (NeuroSleeve) and a custom-made assistive hand exoskeleton for people suffering from hand sensorimotor disabilities (Fig. 1, A to C), whose integration and dynamic use can be seen in movie S1. SensoExo provides an intuitive bidirectional human-machine interface that synchronizes transcutaneous electrical stimulation with robotic assistance (Fig. 1D). It enables precise closed-loop control by delivering somatotopic feedback through TENS to the ulnar and median nerves (Fig. 1C). Simultaneously, it supports exoskeleton movements with coordinated FES-driven activation of the FD and ED muscles, enabling robust hand opening and closing (Fig. 1C). This implementation secures real-time functionality by maintaining a latency of less than 20 ms, which is undetectable to users. The device features a versatile control system, designed to adapt to patient-specific disabilities, accommodating different control inputs (Fig. 1D). These include either implicit (bending sensors, for patients with minimal residual movement) or explicit (manual switches, for patients with complete loss of function) inputs. The bending sensor is located along the dorsal aspect of the finger on the hand module (Fig. 1A). This sensor tracks changes in the finger’s range of motion (RoM), detecting the intention of hand opening and closing through residual finger movement. Otherwise, the user can manually control SensoExo, using switches operated by the contralateral hand.

**Fig. 2.**
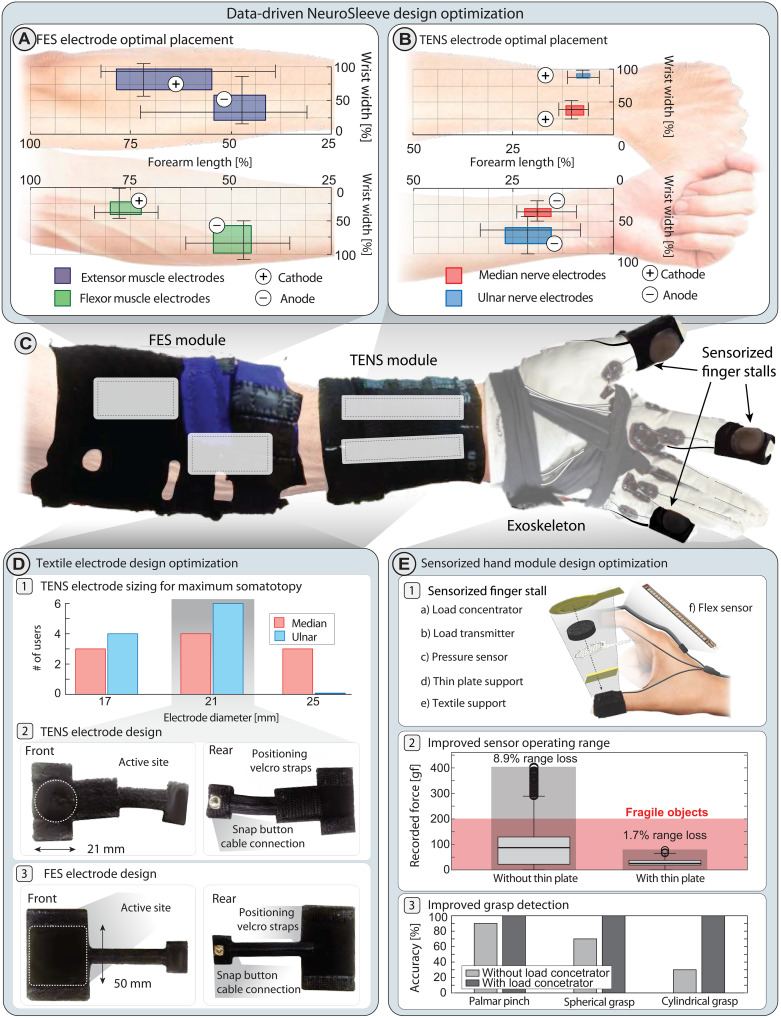
NeuroSleeve data-informed design. (**A**) Bidimensional box plots displaying the optimal locations of anode and cathode for FES of the flexor and extensor finger muscles. (**B**) Bidimensional box plots representing the optimal locations of anode and cathode for TENS of the median and ulnar nerves. (**C**) Graphical representation of NeuroSleeve, with white rectangles indicating Velcro positioning guides for electrode positioning based on (A) and (B). The custom-made sensorized finger stalls (design presented in E1) are positioned over the exoskeleton fingertips. (**D**) 1) Bar plot of the best somatotopy occurrence achieved versus electrode size in healthy participants. 2) Final design of the TENS electrode. 3) Final design of FES electrodes. (**E**) 1) Exploded view of the sensor and its components within finger stalls. 2) Box plot of recorded values from the force sensors while no force is applied, showcasing improved sensor range with the introduction of a thin plate support, which allows it to be sensitive enough to fragile objects. 3) Bar plots showing the increased accuracy of grasp detection during three types of grasping thanks to the introduction of the load concentrator within 10 consecutive grasps.

More in detail, the NeuroSleeve implements three main modules: custom-made sensorized finger stalls worn over/under an exoskeleton (Fig. 1A), a stimulation controller (Fig. 1B), and neurostimulating textile sleeve (Fig. 1C). All of these are seamlessly integrated, within a compact three-dimensional (3D) printed fully portable backpack (Fig. 1D) of approximately 20 cm–by–25 cm–by–5 cm dimensions and weighing 1.5 kg. The system is battery-powered, enabling 6 hours of active use. The sensorized finger stalls serve as the sensing module, comprising three stalls designed to be worn on the thumb, index finger, and little finger. Each stall is equipped with a force sensor strategically placed directly above the fingertips (Figs. 1, A and D, and 2E). These sensors are implemented to detect grasping forces ranging between 100 and 40.000 gf (gram-force). When a touch event occurs, a microcontroller, based on prior subject-specific calibration, linearly modulates the stimulation in real-time using the sensor readings. Depending on the user’s impairment, FES and/or TENS stimulation is applied through two custom-made neurostimulating modules to enhance motor and/or sensory function. Both FES and TENS are pulse-width modulated, with patient-specific minimum and maximum pulse widths defined during the characterization phase. NeuroSleeve is designed to be compatible with various exoskeleton types, ensuring adaptability to different assistive needs. To validate this, we constructed two customized types of SensoExos (Supplementary Methods): by modifying RELab tenoexo (RELab, ETH Zurich, Switzerland) and by designing a specifically designed CATCHGlove (ARIES Lab, Heidelberg University, Germany) (fig. S1). These two devices have shared control with NeuroSleeve and provide a custom-made modular hand unit (Fig. 1D), assembled through the integration of hard and soft materials, offering enhanced support for hand opening in severe spastic cases where there is no finger movement. In addition, the combined use of these hand modules and electrical stimulation offers a quicker and more effective solution to relax spastic finger muscles compared to traditional cold exposure treatments (*78*), significantly reducing the time and complexity of preparing for hand module fitting. This reduced donning and doffing times by ~50% (measured in most five spastic patients) compared to standard gloves that can take more than 10 min. Furthermore, the simultaneous use of FES and the exoskeleton for hand assistance led to a 30% reduction in motor current demand during repetitive tasks, suggesting an important extension of the device’s battery life.

### NeuroSleeve data-informed design

We designed the NeuroSleeve garment using a data-driven approach informed by trials with 10 healthy participants (Fig. 2). This process allowed us to determine the optimal electrode positions and sizes: Preliminary experimental results informed subject-specific adjustments to electrode placement, ensuring a more flexible design. In addition, these results guided improvements to the reliability of resistive flexible force sensors placed directly under the fingertips for touch detection. To address signal distortion caused by sensor compliance, we introduced purposefully designed structures that enhanced measurement accuracy during hand motion and grasping, resulting in more consistent stimulation.

#### Selecting the optimal FES module electrode placement

We determined the optimal electrode placement for the FD and ED muscles using the results from the healthy volunteers’ motor characterization test. We identified the normalized distribution of the best electrode configurations that were able to elicit finger flexion and extension while minimizing wrist flexion and extension (Supplementary Methods). Positions were normalized on the basis of anthropometric measurements (i.e., arm length and wrist width). Analysis of the data revealed that both muscle groups exhibited two major clusters for the optimal placement of the anode and cathode electrodes (Fig. 2A). The anode position for both muscles was consistently identified at ~50% of the forearm length (50.4 ± 15.1% for ED, 48.5 ± 7.8% for FD). Conversely, the cathode was positioned more proximally, around 70% of the forearm length (66.2 ± 15.4% for ED, 79.3 ± 5.1% for FD). Optimal anode and cathode positions along the horizontal axis of the arm are found contralaterally with respect to the sagittal plane of the forearm (40.3 ± 24.9% of wrist width for ED anode, 82.6 ± 25.4% for FD cathode, 78.4 ± 20.9% for FD anode, and 28.0 ± 18.7% for FD cathode). The anode-cathode orientation is aligned with the anatomical longitudinal disposition of the FE and ED muscle groups.

The obtained optimal electrode placements, represented by a checkerboard pattern, revealed a symmetrical positioning of the electrodes along the longitudinal axis. This allowed us to design the NeuroSleeve so that it can be used interchangeably for both the left and right sides of the body creating specific Velcro positioning guides for the electrodes placed according to Fig. 2C. These guidelines served to anchor the electrodes to the inner part of the sleeve and guide the electrode placement when the sleeve is worn.

#### Selecting the optimal TENS module electrode placement

In a similar manner to FES stimulation, we performed a sensory characterization test of different preselected TENS electrode sizes (Supplementary Methods). In Fig. 2B, we show the results of this calibration as a normalized distribution with respect to participant anthropometric measurements (i.e., arm length and wrist width). These distributions showed higher variability in covered surface along the longitudinal axis over the trajectory of the nerves (18.7 ± 4.5% of the forearm length for median anode, 9.9 ± 2.6% for median cathode, 21.5 ± 8.3% for ulnar anode, and 7.9 ± 2.3% for ulnar cathode) for both the anode and cathode placements (Fig. 2B). The anode and cathode were placed at the same distance with respect to the horizontal axis of the arm (37.7 ± 11.9% of the wrist length for median anode, 38.2 ± 9.9% for median cathode, 68.9 ± 18.5% for ulnar anode, and 86.8 ± 12.5% for ulnar cathode), meaning that the anode and cathode were, on average, aligned with the sagittal axis (Fig. 2B). The obtained results were used to design two parallel Velcro positioning guides for the TENS electrodes, with the same purpose they had for the FES module as shown in Fig. 2C. This design ensured that the TENS sleeve was symmetrical and could be used interchangeably for both the right and left hands.

#### Selecting the optimal size of TENS and FES electrodes

To ensure effective artificial sensory restoration, it was essential to minimize sensations perceived directly beneath the stimulation electrodes, as these can interfere with somatotopic perception in the hand. Although gel-based commercial electrodes provide low skin-electrode impedance under static conditions, their use within a wearable sleeve posed challenges during prolonged use and repeated donning and doffing. Gel dehydration and redistribution over time led to spatially inhomogeneous skin-electrode contact and current density, increasing the occurrence of nonsomatotopic sensations localized under the electrode. To address these limitations, we introduced conductive textile electrodes (Supplementary Methods), which provided more stable and reproducible stimulation. This design reduced uncomfortable under-electrode sensations while improving wearability, hygiene, and reusability, thereby enhancing user comfort and usability during sensory restoration. Second, we investigated the quality of the elicited sensation based on different sizes. The 17- and 21-mm-diameter TENS electrodes elicited the largest area of sensation in the hand, covering an average of 100 ± 22.7% of the fingers innervated by the median nerve and 56.2 ± 34.3% of the fingers innervated by the ulnar nerve. However, when evaluating the quality of the artificial sensation, most participants expressed a higher preference for sensation quality (40 and 60% of the participants, for median and ulnar nerves, respectively) for the 21-mm electrode size (Fig. 2D). Unlike the TENS electrodes, FES patches, thanks to their larger surface area, did not exhibit uncomfortable artificial sensations beneath the electrode. Consequently, we designed the electrode for FES patches using the same conductive textile material matching in size to the commercial counterparts (50-mm width by 50-mm height) (Fig. 2D).

#### Improving finger stalls sensors force detection

We developed sensorized finger stalls that could be easily worn under or over the exoskeleton. To record the force exchange during the grasp, we embedded resistive force sensors placed over the fingertip (Fig. 2E1). However, the high flexibility of the chosen sensors, combined with the deformations caused by strapping them around the fingers, resulted in a preloading effect on the sensors. This preloading effect caused a loss of functional range for low-level forces, amounting to 8.9%, equivalent to approximately 400 gf. To mitigate this issue, we introduced the supportive 3D-printed thin plate, which reduced the loss of operating range to below 1.7% (29.0 ± 11.6 gf) (Fig. 2E2). This improvement in range retention ensures more accurate and reliable force measurements, especially during grasping of fragile objects (<200 gf).

In addition, the force sensors, due to the small force sensing areas (<10 mm), exhibited reduced performance in detecting forces during grasping. Specifically, the detection rates were 90% for palmar pinch, 70% for spherical grasp, and 30% for cylindrical grasp during testing with a healthy subject. However, the inclusion of custom-made 3D-printed load concentrators and foam load transmitters significantly improved grasp detection to 100% accuracy in all types of grasping (Fig. 2E3).

### Clinical testing of SensoExo system

The SensoExo system was tested on a total of 14 participants with neurological impairments. The electrical stimulation provided by NeuroSleeve was customized to meet each participant’s level of disability.

Since all participants reported sensory deficits during the medical assessment, the NeuroSleeve TENS module was included for everyone (S01 to S14) to provide artificial sensory feedback. In contrast, the FES module was only introduced for participants with severe motor impairment (*N* = 7: S01, S03, S07, S08, S11, S12, and S13), who had less than 50% of their normal finger RoM and were unable to effectively activate finger muscles. These individuals could not properly use the flex sensor for intention detection, requiring FES to facilitate movement, and therefore controlled the SensoExo via a button-based system operated with the contralateral hand. Participants with greater residual mobility (*N* = 7: S02, S04, S05, S06, S09, S10, and S14) maintained sufficient voluntary control to operate the device using the flex sensor alone, making FES unnecessary and disruptive to their refined control.

The protocol included three types of tests: sensory characterization, motor characterization, and functional tasks (grasp and release). Each test was performed under three conditions: (i) “unassisted,” (ii) assisted with a soft “exoskeleton alone,” and (iii) “SensoExo” (exoskeleton plus NeuroSleeve). All trials and conditions were conducted in a randomized order.

Of the 14 participants who completed the characterization phase with the SensoExo system, 8 participants (S04, S06, S08, S10, S11, S12, S13, and S14) additionally completed the functional grasp-and-release evaluation, whereas the remaining participants did not proceed due to health-related issues or inability to complete the task (further details on participant flow and provided support are reported in table S1).

### NeuroSleeve improves motor and sensory functions with respect to standard assistive orthoses

**Fig. 3.**
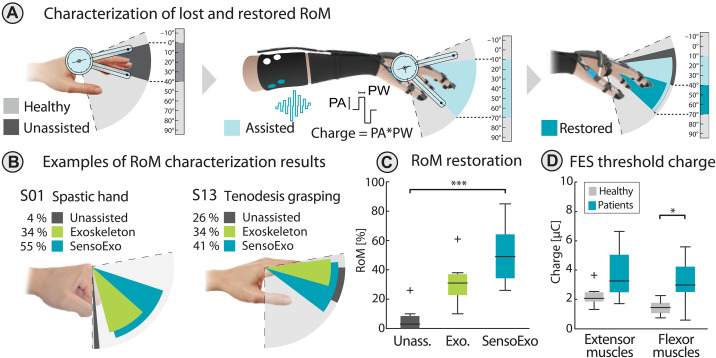
SensoExo improves RoM in people with hand impairment. (**A**) Methodological approach and definition for calculating restored RoM: Using a goniometer, we characterized the RoM during both the unassisted (dark gray) and assisted (light blue) conditions at the MCP joint of the hand. Light gray represents the RoM of a healthy individual’s MCP joint. The restored RoM (dark blue) through assistance (exoskeleton alone or SensoExo) is defined as the difference between the assisted condition and the unassisted RoM. (**B**) Examples of characterization results for S01 (spastic hand) and S14 (tenodesis grasp). In dark gray, the baseline (impaired) RoM is shown. In green, the RoM with the use of the exoskeleton, and in dark blue, the RoM with SensoExo (exoskeleton with FES) is displayed. (**C**) Box plot of the RoM comparison for the participants with higher motor impairment (<50% of the healthy RoM, *N* = 7: S01, S03, S07, S08, S11, S12, and S13) who performed under unassisted, exoskeleton alone, and SensoExo conditions. Statistical differences, determined using the Friedman test with Bonferroni post hoc correction for multiple comparisons, are displayed. (**D**) Box plot comparing the charge threshold for eliciting finger movement between eight healthy volunteers and seven subjects who underwent SensoExo characterization. Mann-Whitney-Wilcoxon test results are displayed. *P* values legend: *P < 0.05 and ***P < 0.001.

**Fig. 4.**
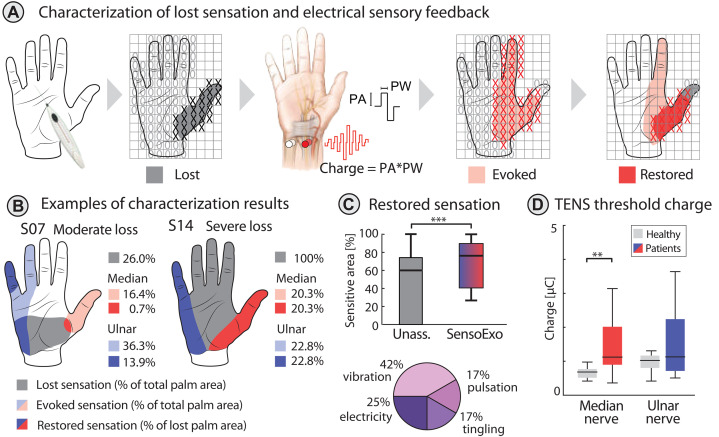
SensoExo improves tactile feedback in hand impairment. (**A**) Through a QST test, we first characterized the impaired sensory area (dark gray), and following, we quantified the area of the evoked artificial sensation (light red). The dark red area represents the restored area of sensory feedback with respect to the baseline, obtained when the TENS stimulation by the SensoExo was active. (**B**) Examples of characterization results for two subjects, S07 (moderate sensory loss) and S14 (severe sensory loss). In dark gray, the initial lost area of sensation is shown. In light red and blue, the artificially elicited sensation respectively obtained through median and ulnar stimulation. In dark red and blue, the artificially restored sensation respectively obtained through median and ulnar stimulation, that is, the intersection between the lost and the evoked maps. (**C**) Box plot comparing the total hand area in which sensory feedback was perceived across all subjects between the unassisted and the SensoExo condition. The pie chart summarizes the qualitative nature of the evoked sensations reported by participants. Statistical differences, determined using the Wilcoxon signed rank test, are displayed. (**D**) Box plot comparing the charge threshold for eliciting minimum artificial sensation in eight healthy volunteers and the subjects (*N* = 14). Mann-Whitney-Wilcoxon test results are displayed. *P* values legend: **P < 0.01 and ***P < 0.001.

We initially conducted two separate assessment sessions for all recruited patients: a motor characterization test to evaluate their motor capabilities and a sensory characterization test to assess the extent of their sensory perception. These assessments included measuring hand RoM (Fig. 3) and mapping the sensitive palm area (Fig. 4), both performed under unassisted and assisted conditions.

As stated in the previous section, participants with more than 50% of healthy finger RoM (*N* = 7: S02, S04, S05, S06, S09, and S10) did not receive FES support during the SensoExo condition, as FES disrupted their natural grasp by interfering with voluntary muscle control. The 50% RoM loss threshold was defined a priori based on clinical expertise. An exploratory post hoc analysis revealed significant impairment-dependent differences in baseline performance, suggesting that this classification reflects meaningful differences in impairment profiles. As a result, during the motor assessment, the SensoExo condition provided no additional motor benefit for these patients compared to the exoskeleton alone, making the latter condition redundant. Therefore, these participants were not tested under the SensoExo condition for RoM evaluation.

For sensory assessment, since the soft exoskeleton does not provide any sensory tactile restoration, the exoskeleton alone condition was omitted from the protocol. Testing was conducted under the unassisted condition and SensoExo which evaluated the effect of artificial sensory feedback.

#### FES module enhances RoM restoration when coupled with assistive exoskeleton

To evaluate and compare the impact of the SensoExo assistance, we measured the metacarpophalangeal (MCP) RoM under both unassisted and assisted conditions. The difference in RoM between these conditions indicates the degree of restored healthy range of the fingers’ mobility (Fig. 3A).

This motor evaluation showed substantial limitations in the provided motor assistance of the exoskeleton alone, especially when participants experienced spasticity or relied on tenodesis grasping (Fig. 3B). For instance, subject S01, who has severe hand spasticity, characterized by a tightly clenched fist and involuntary muscle tightness (total baseline RoM: 4%), showed limited improvement with the exoskeleton alone (34% of healthy RoM). In contrast, the combination of FES and exoskeleton restored 55% of the healthy range, demonstrating the added benefit of coupling soft robotics with electrical stimulation. Conversely, when patients completely lose motor finger abilities (e.g., patient S13), they can be trained in using tenodesis grasping (*79*), a passive functional hand grasp triggered by the finger flexors’ shortening from wrist joint extension. In the unassisted condition, this technique enabled the patient to achieve 26% of the healthy RoM. With the combined assistance, the system increased finger RoM to 41% of the healthy range, while the exoskeleton alone provided only limited improvement, reaching 34%.

Using data from the subset of participants who were able to test both assistance conditions (those with less than 50% residual RoM), we assessed the overall impact of adding FES into the SensoExo system. For these seven participants, who presented a severely reduced baseline RoM (6.29 ± 9.81% of the healthy range), the SensoExo system (exoskeleton plus FES) achieved a significantly higher RoM (51.71 ± 20.99%) than the exoskeleton alone (32.00 ± 15.67%) (Fig. 3C). Across these three conditions (no support, exoskeleton only, and SensoExo), we observed a significant overall effect (*P* < 0.001). Multiple comparisons confirmed a statistically significant improvement when SensoExo was used compared to no support (*P* < 0.001), although the difference between the other conditions did not reach statistical significance (*P* = 0.061). For participants with less severe motor loss, results were more variable. Subject S04 showed a slight improvement with the exoskeleton alone, while subjects S09 and S14 saw no change in impaired RoM under exoskeleton-only conditions (fig. S2). Subjects S06 and S10 had no motor impairment.

Furthermore, we compared the charges required to reach a participant motor threshold (finger motion). In impaired subjects, it was higher for both the extensor (3.86 ± 1.94 μC) and flexor muscles (3.16 ± 1.69 μC) than in healthy participants (0.68 ± 2.22 μC and 0.5 ± 1.45 μC, respectively) (Fig. 3D). A statistically significant difference was observed specifically for the flexor muscles (*P* = 0.042), while no significant difference was found for the extensor muscles. Participant-specific FES parameters (i.e., amplitude and maximum and minimum pulse width) for finger flexor and extensor muscles are listed in table S2.

#### TENS module enhances somatosensory feedback in the volar area of the hand

Participants’ sensory loss was assessed through quantitative sensory testing (QST) using a 10g Semmes-Weinstein monofilament to identify areas with complete loss of protective sensation (*80*). We then compared these insensitive regions to areas where TENS applied to the median and ulnar nerves could evoke artificial sensations. If participants could feel this artificial tactile feedback in regions previously unresponsive to the monofilament, we considered the sensation to be restored (Fig. 4A). In cases of moderate sensory loss (e.g., S07), TENS restored sensation to more than half of the previously unresponsive areas (26% of the palm). Even when the loss was complete (e.g., S14), TENS still restored 43.1% of the insensitive area (Fig. 4B). These results showed that SensoExo, if coupled with electrical stimulation, can provide meaningful tactile feedback to the fingers and palm, regardless of the severity of sensory impairment. In most participants, we successfully restored lost areas of the palm and digits that were innervated by the ulnar and median nerves (fig. S3). However, participants S08, S05, and S06 likely had more severe central neural damage. They could only perceive artificial sensations from one of the two nerves, not both. Participants S09 and S12 showed no sensory loss at all.

When comparing the sensitive area of the hand, we observed a significant difference (*P* < 0.001) between the condition without support (47.35 ± 39.95% of the palm area sensitive) and the TENS-assisted condition (66.49 ± 27.65%), as shown in Fig. 4C. A comparison between hand regions of sensory loss and regions in which somatotopic sensations were restored through median and ulnar TENS stimulation across all participants is additionally presented in fig. S4. We also examined the quality of these artificial sensations (Fig. 4C). Most participants (42%) described the feeling as vibration-like, while 25% reported an electric sensation. The remaining participants were evenly split between describing pulsation and tingling. In addition, we compared the sensory thresholds. Last, we compared sensory thresholds. For median nerve stimulation, participants with impairments had significantly higher thresholds (1.42 ± 0.81 μC) than healthy participants (0.69 ± 0.18 μC) (*P* = 0.006) (Fig. 4D). For ulnar nerve stimulation, there was no significant difference in the minimal charge required to evoke a localized sensation between impaired and healthy participants (1.43 ± 0.99 μC versus 0.91 ± 0.26 μC), although the impaired participants demonstrated higher variability. Participant-specific TENS parameters (i.e., amplitude and maximum and minimum pulse width) for median and ulnar nerve are listed in table S2.

### SensoExo provides tailored assistance for different object types based on subject impairment

**Fig. 5.**
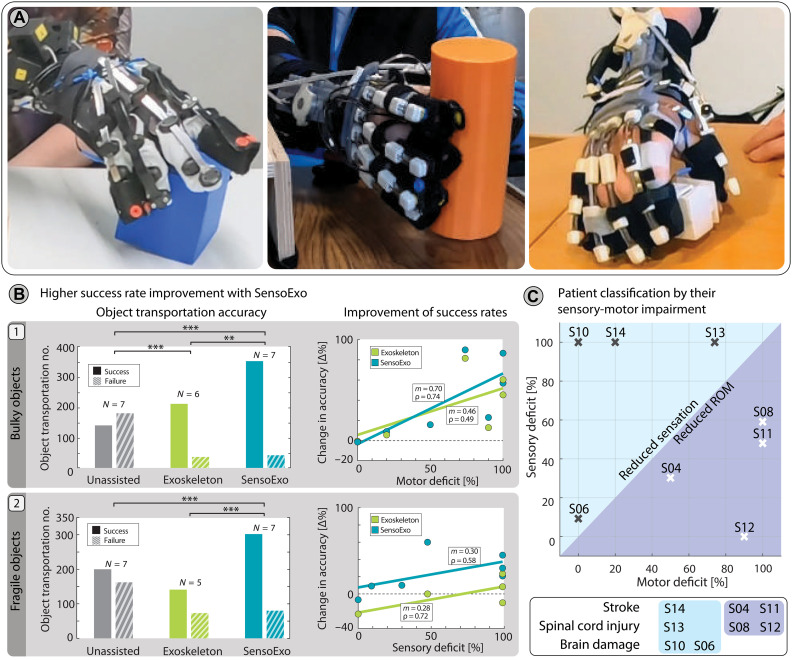
SensoExo provides tailored assistance based on patients’ impairment. (**A**) Examples of successful grasping of the cube, the cylinder, and the virtual egg (from left to right) during the GRT using SensoExo with the two types of portable assistive exoskeletons. (**B**) Changes in success rate of the GRT across the three testing conditions (unassisted, exoskeleton, and SensoExo), grouped per bulky and fragile objects. In (B1), bulky objects, the bar plot illustrates the number of succesfully transported objects (plain) during the GRT for each condition, with respect to the failed ones (striped). The variable “*N*” represents the number of subjects performing the specific condition. The scatterplot shows the change in GRT accuracy versus subjects’ motor loss with bulky objects between the unassisted and both the assisted (exoskeleton and SensoExo) conditions. The linear regression between these two datasets is depicted along with the corresponding ρ value. In (B2), fragile objects, the bar plot illustrates the number of accomplished transported objects (plain) during the GRT for each condition, with respect to the failed ones (striped). The variable *N* represents the number of subjects performing the specific condition. The scatterplot displays the change in GRT accuracy versus subjects’ sensory loss with fragile objects between the unassisted and both the assisted (exoskeleton and SensoExo) conditions. Pairwise chi-square test results are displayed. *P* values legend: **P < 0.01 and ***P < 0.001. The linear regression between these two datasets is depicted along with the corresponding ρ value. The *P* values of the chi-square test results adjusted with Bonferroni correction are reported for the bar plot. (**C**) Participants are categorized into two groups based on the severity of their sensory and motor loss, irrespective of the type of disability they have.

When suffering from motor and sensory impairment, participants have difficulties in performing even a simple ADL like drinking from a bottle (*81*). While the exoskeleton alone was not sufficient to help participants complete this task, the combined SensoExo system enabled them to do so with high accuracy (see movie S2). To further investigate the functional benefits of SensoExo, a subsequent GRT session was conducted. Eight of the 14 participants performed GRT tests (table S1). Each participant performed 30 trial repetitions with four different objects representing real-world scenarios: two bulky objects (a cube and a cylinder) and two fragile objects (virtual eggs with breaking points at 200 and 100 gf) (Fig. 5A).

When handling bulky objects (Fig. 5B1), participants achieved their highest success rate using SensoExo (90.03%, number of trials = 391), outperforming both the unassisted condition (43.75%, number of trials = 320) and the exoskeleton alone (85.43%, number trials = 247). Pairwise chi-square test showed significant statistical difference among all the conditions (*P* < 0.001). We subsequently applied a binomial generalized linear mixed-model (BGLMM) to correct the chi-square outcome, accounting for the unique participant identifiers, the testing conditions, and the trial sequences as predictors. The model revealed significant participant clusters (*P* < 0.001) and confirmed a significant effect in the testing condition (*P* < 0.001) but no notable trial sequence influence (*P* = 0.18), excluding learning effects during the repetitions. Multiple comparisons with Bonferroni correction validated chi-square pairwise findings (SensoExo versus exoskeleton alone *P* = 0.005, rest *P* < 0.001). Moreover, SensoExo provided greater performance improvements relative to the unassisted conditions, especially for participants with more than 50% motor loss. A linear regression relating motor deficit to accuracy improvement showed a steeper slope for SensoExo (*m* = 0.70, ρ = 0.58, *P* = 0.056) than for exoskeleton alone (*m* = 0.46, ρ = 0.49, *P* = 0.41), indicating larger gains for those with severe motor impairments.

A similar pattern emerged with fragile objects (Fig. 5B2). The SensoExo condition again yielded the highest success rate (79.06%, number trials = 382), outperforming the other two conditions (respectively 65.58%, number trials = 215 and 55.25%, number trials = 362). Pairwise chi-square test yielded statistically significant differences between the testing conditions. When correcting the output for the BGLMM, we found again significant participant clusters (*P* < 0.001), a significant testing condition effect (*P* < 0.001), with no notable trial sequence effect (*P* = 0.19). Multiple comparisons for the testing condition however showed significant differences among conditions (*P* < 0.001) except for unsupported and exoskeleton-only condition (*P* = 1.00). Examining changes relative to no support, exoskeleton-only assistance was even detrimental in the functional outcome when the sensory loss is minimal (<50%, *m* = 0.28, ρ = 0.72, *P* = 0.168). In contrast, the SensoExo demonstrates the ability to provide higher success rate as the degree of sensory deficit increases (*m* = 0.30 ρ = 0.57, *P* = 0.173).

Building on the positive effects observed across participant clusters, we analyzed general trends in their behavior. Participants displayed a wide range of sensorimotor deficits, even within the same condition (Fig. 5C and table S1). Notably, there was no significant correlation between sensory and motor impairments (*R* = −0.21, *P* = 0.62). Instead, participants tended to cluster based on their dominant impairment type: motor or sensory (Fig. 5C). The motor-impaired group (S04, S08, S11, and S12) exhibited higher motor deficit (85.00 ± 23.81% of the RoM), leading to significant issues with grasp, transport, and release tasks involving the bulky objects (35.44 ± 40.95% unassisted success rate). Using SensoExo, this cluster of participants demonstrated consistently higher success rates (80.53 ± 16.58%), compared to the exoskeleton alone (63.33 ± 20.21%). Specifically, S11 and S12 showed significant improvements (*P* < 0.001, *P* = 0.004), while S08 showed no difference (*P* = 0.119). S04 did not complete the exoskeleton-only condition (fig. S5). The sensory-impaired group (S06, S10, S13, and S14) had exhibited instead a substantial sensory loss (77.31 ± 45.37% of palm area) and struggled the most with fragile objects (60.63 ± 10.35% unassisted success rate). With SensoExo, their success was consistently higher (83.98 ± 5.25%), when compared to the exoskeleton alone (59.80 ± 17.67%). S10, S13, and S14 showed statistically significant differences between the two assisted conditions (*P* < 0.01, *P* = 0.15, and *P* = 0.02, respectively). S06 did not perform the GRT task with the exoskeleton for the fragile objects.

### Participants adapt their grasp-and-release strategy while provided with sensory and motor assistance

**Fig. 6.**
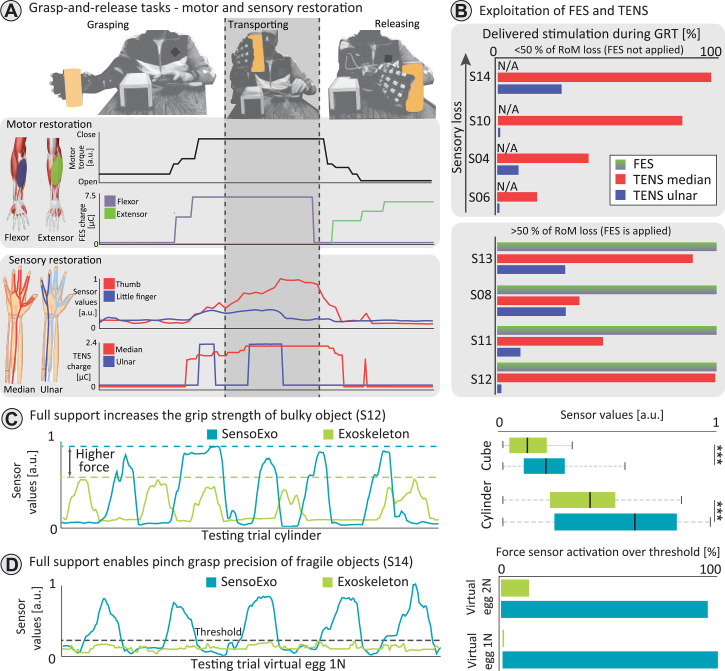
Artificial sensory feedback exploitation in patients during GRT. (**A**) Illustration of electrical stimulation recordings during GRT. In the motor restoration section, the flexor FES (purple) synchronizes with the exoskeleton’s closing motion, while the extensor FES (green) is active during the exoskeleton’s opening phase. In the sensory restoration section, force sensors capture the grasping force, guiding the modulation of electrical charge for median TENS (red) and ulnar TENS (blue). This stimulation enables somatotopic sensation restoration in the thumb and pinky, where the actual force is applied. (**B**) Bar plots presenting the percentage of stimulation delivery for all subjects during GRT. Participants are categorized on the basis of their motor impairment and ordered by sensory impairment; for participants with motor impairment below 50%, FES was not used and therefore marked as not applicable (N/A). The graph displays FES in green and purple, TENS for median nerve in red, and TENS for ulnar nerve in blue. (**C**) Example of FES use by S12 during GRT with large objects on the left. The subject exerts higher force on the sensor only during SensoExo support. The sensor value (a.u., arbitrary units) distribution during the trial is shown on the right, highlighting the higher forces due to the introduction of FES during SensoExo condition. Results of Wilcoxon signed rank test are displayed. *P* values legend: ***P < 0.001. (**D**) Example of TENS exploitation of S14 during tasks involving the grasp and release of fragile objects (left). The sensor values for both the exoskeleton-only (green) and during SensoExo (blue) conditions are depicted. During SensoExo support, the subject applies force specifically over the sensors, while this does not happen in the exoskeleton-only condition. The accompanying bar plot (right) illustrates the percentage of instances when the subject applies force on the sensors during the fragile object manipulation.

Integrating FES and TENS into the SensoExo system allowed the exoskeleton to provide both motor assistance and sensory feedback. By coordinating the exoskeleton’s motors with charge delivered to the flexor and extensor muscles stimulation, we enabled users to achieve continuous and more precise modulation of the grasping force (Fig. 6A). At the same time, calibrated TENS stimulation, based on sensor reading, to the median and ulnar nerves provided modulated artificial sensation, helping users adjust their grip force.

To understand how participants used this sensory feedback, we analyzed the frequency of TENS stimulation delivered during successful GRT trials. We found that participants with more severe sensory deficits relied on TENS more often (Fig. 6B, top). Participants predominantly used median nerve stimulation (64.42 ± 31.48%), restoring thumb sensation with respect to ulnar stimulation (14.7 ± 15.53%) and restoring little finger sensation (Fig. 6B, top). FES was applied only to those who lost more than 50% of their healthy RoM, and when provided, participants used it in all successful trials (Fig. 6B, bottom).

To quantitatively assess how these different forms of assistance influenced functional performance, we analyzed GRT success using a BGLMM. The model accounted for the type of support (no support, exoskeleton alone, and SensoExo), the impairment profile (predominantly motor versus sensory), and the task type (bulky versus fragile objects) while controlling for intersubject variability by including participant identity as a random effect. The model revealed a large and statistically robust main effect of support type on task success (*d*_z_ = 1.39, 95% confidence interval [1.10, 1.68], *P* < 0.001), confirming that SensoExo significantly improved performance compared to the other conditions. Moreover, significant SupportType×TaskType and ImpairmentType× TaskType interactions (*P* < 0.001) demonstrated that performance gains were not uniform but depended on both task demands and the participants’ impairment profile. These interaction effects strongly support the observation that participants adopted different strategies when using the SensoExo system, selectively exploiting sensory or motor assistance according to task requirements. We further analyzed functional behavior, looking at the recorded sensor data. For instance, when carrying large objects requiring strong and stable forces, participants with predominant motor impairments preferentially relied on FES support. Specifically, during the GRT with the cube and cylinder, participant S12 used FES stimulation to significantly enhance grip force compared to the exoskeleton alone, as shown by higher force sensor values while transporting both objects (*P* < 0.001; Fig. 6C). This improvement is demonstrated in movie S3. In contrast, when handling fragile objects, precise tactile feedback played a dominant role. Without TENS, participant S14 showed no measurable force adjustments on the fingertips while manipulating delicate virtual eggs (13.3 and 0% of the time for the two types of virtual eggs respectively; Fig. 6D) using the exoskeleton alone. This suggests reliance on other cues, such as vision rather than touch. With SensoExo, however, force sensors registered significant force changes during grasping (96.4 and 100% of the times for the two types of virtual eggs respectively), demonstrating that the participant actively exploited the restored sensory feedback to fine-tune force control. TENS helped the participants avoid excessive force and reduced the need for other compensatory strategies, as can be seen in movie S4.

## DISCUSSION

### Modular NeuroSleeve system can provide additional sensorimotor assistance with different hand exoskeletons

In this study, we developed a data-driven, designed neurostimulating garment for grasping assistance in people suffering from sensorimotor disabilities that could be coupled with two different portable assistive exoskeletons (Fig. 1). Commencing with the initial assessments on healthy participants, we successfully minimized the number of components, resulting in a modular NeuroSleeve capable of providing both motor assistance and sensory feedback restoration (Fig. 2, A and B). TENS and FES components were designed with specific positioning guidelines for electrode placements to enhance wearability (Fig. 2C). Moreover, we systematically defined the optimal electrode size for both TENS (21-mm diameter) and FES (50-mm length) for comfortable and effective stimulation (Fig. 2D). For the sensing module of NeuroSleeve, we tailored finger stalls with force sensors, increasing the grasp recognition from 63.33 ± 30.55% up to 100% in bench-level validation tests across the evaluated grasp types. The changes in design demonstrated significant improvement of sensitivity to about 30 gf, crucial for the manipulation of fragile objects (Fig. 2E).

We designed NeuroSleeve to enhance electrical stimulation versatility and reliability. This effort was crucial for the development of an assistive device capable of addressing the notable heterogeneity among individuals experiencing sensorimotor impairments. In addition, NeuroSleeve was designed with the purpose of being seamlessly integrated with assistive exoskeletons, thereby augmenting the level of offered assistance. This distinctive attribute sets it apart from other existing devices, such as Intento (Lausanne, Switzerland) or NeuroLife (Battelle, Ohio, USA) sleeves. However, the paramount advantage of NeuroSleeve, in contrast to existing alternatives, lies in its capability to restore both motor and sensory feedback.

### SensoExo enhances sensorimotor functions in participants with hand impairment

In the initial assessment session, the SensoExo demonstrated higher sensorimotor assistance in participants compared to the exoskeleton alone (Figs. 3 and 4). SensoExo provided ~20% greater restoration of the healthy RoM compared to the exoskeleton alone. This improvement is likely attributed to the NeuroSleeve’s FES module capability to directly activate muscular contractions, even in the presence of atrophied and spastic muscles, that may resist externally applied mechanical forces (*82*, *83*). The electro-neural activation of the muscles, bypassing the damage to the central nervous system, empowered each user to apply additional joint torque synchronously with the exoskeleton, thereby increasing the restored joint RoM in all participants (fig. S2).

In addition, sensory assessments revealed that the NeuroSleeve’s TENS module could artificially restore touch-like sensations in ~20% more of the previously insensitive palm area, on average. Notably, this artificial restoration was achieved regardless of the severity of the participants’ sensory deficit (fig. S3). This finding suggests that TENS-based assistive device has the potential to induce sensory restoration even in cases where the loss is due to damage to the central neural region. This approach may help address previous challenges of intense cognitive load associated with the use of remapped vibrotactile sensations (*84*).

### SensoExo produces tailored assistance during functional tasks

During functional tasks, participants performed the GRT with two different sets of objects that challenged their remaining grasping abilities. With bulky objects, participants achieved a higher success rate using SensoExo compared to the exoskeleton alone (Fig. 5B1). Across all subjects, SensoExo provided greater functional improvements, especially for those with more limited mobility. Similarly, when handling fragile objects, SensoExo achieved greater accuracy (Fig. 5B2). In contrast, using the exoskeleton alone offered little or no improvement over the unassisted condition. Overall, SensoExo consistently delivered statistically superior functional performance compared to no assistance.

Notably, we found no correlation between participants’ levels of sensory and motor deficits. Instead, participants could be grouped on the basis of their primary type of impairment (Fig. 5C). Those with more severe motor deficits benefited the best from SensoExo assistance when transporting bulky objects, achieving higher success rates (fig. S5A). These findings align with recent research in assistive applications for both upper and lower limbs, underscoring the heightened effectiveness of robotic wearable devices when complemented by FES (*85*, *86*). Conversely, participants with pronounced sensory loss showed consistently better performance with SensoExo when handling fragile objects (fig. S5B). This outcome suggests that restoring targeted, somatotopic sensory feedback in individuals with central nervous system damage can yield improvements comparable to those observed in amputees (*84*).

### The mechanisms underlying SensoExo’s effectiveness

Looking at the overall results, the integration of the NeuroSleeve system with the portable assistive exoskeleton (SensoExo) has proven to be highly beneficial in providing both motor support and sensory feedback for individuals with hand impairments. The coordinated use of FES with the exoskeleton motor output force allowed participants to achieve a more robust and controlled grasp. From investigating the recorded data, we could see that participants were exerting significantly higher additional force on the object when FES was applied (Fig. 6D and movie S3). Movement precision and fine force control, on the other hand, rely mostly on the sensory feedback provided by TENS. Users intentionally exploited the provided sensory feedback 98.2 ± 1.8% of the time adapting preexisting compensatory strategies to avoid the object breakage (Fig. 6C and movie S4). While acknowledging SensoExo proof-of-concept status and its limitations in terms of everyday reliability, our findings emphasize its effectiveness as an assistive device. SensoExo enabled participants to handle delicate items while also enhancing the force applied for heavier and larger objects.

### Limitations

The major limitation of this study was found in the consistency of the number of trials per subject. Because of the delicate health and psychological condition of the participants, various interruptions to the protocol were necessary. Frequent occurrences of spasms, pain, fatigue, lethargy, and low motivation required us to pause the study intermittently. Consequently, the experiments were carefully adjusted to prevent any demoralization among the participants and to safeguard the overall integrity of the session’s outcomes. Furthermore, the occasional emergence of comorbidities compelled us to terminate the study prematurely for 6 subjects of the initial 14 (S01, S02, S03, S05, S07, and S09). Some participants did not sustain the functional test due to health reasons or due to impossibility of completing the functional tasks, which resulted in an incomplete series of trials for grasp and release or, in some cases, a complete absence of performance in these tasks. Participants’ summary table with complete tests can be found in table S1. This limitation, together with the mixed-etiology composition of the cohort, limits the generalizability of the findings. Although our model-based analysis indicates a large and statistically robust effect even under these conditions, the present results should be interpreted as early evidence. Future studies with larger and pathology-stratified cohorts will be necessary to confirm these findings and better characterize impairment-specific effects.

Furthermore, although the sleeve design was optimized for wearability, the calibration process still occasionally required up to 15 min. One contributing factor was the need for manual adjustments of the electrodes when somatotopy was not immediately achieved. This adjustment was often necessary due to electrode shifting during the donning and doffing of the system. To address this issue, future embodiments of the NeuroSleeve could use an electrode array that allows for the selection of the active site without the need for manual adjustments. In addition, to further expedite the calibration time, the process could be automated by using a smart calibration algorithm (*87*). Such an algorithm would enable automated fine-tuning of the system without direct expert intervention. By eliminating the need for frequent manual interventions, this advancement could streamline the calibration process substantially. Although patients did not report any changes in evoked sensations during the 3-hour sessions, automated monitoring of stimulation stability would enhance system performance. By observing the interface impedance in real time, the system could either alert the user or automatically adjust the stimulation parameters to maintain the consistent intensity of the restored sensation during system usage.

### SensoExo exploitation for assistance of hand impaired participants

The demonstrated approach used by the SensoExo system, encompassing both sensory feedback and motor support, appears to provide means for participants to address the unique challenges posed by their specific impairments. This bidirectional approach sets our technology apart from standard assistive robotic devices and wearable garments that primarily emphasize motor enhancement (*19*, *39*, *47*, *51*). The modular data-driven design of the NeuroSleeve, purposefully intended to target both sensory and motor needs, renders the device adaptable to subjects with different ranges of sensorimotor deficits. Furthermore, the potential for integration with existing assistive portable exoskeletons could open the door to a potentially more effective, and personalized daily assistance. Moreover, our solution delivers sensory and motor improvements comparable to invasive neurotechnologies, without the inherent risks of complex, multisite implant surgeries (*61*, *62*). Given the high surgical burden and associated risks, such invasive interventions are not recommended for frail populations, such as stroke survivors. Our approach thus provides a safer, noninvasive alternative that bridges the gap between functionality and clinical feasibility.

While this study covers only a small portion of the different epidemiology of patients with hand sensorimotor disabilities, we showed the system’s capability to cater to different levels of sensory and motor deficit, allowing participants to improve their hand functionality based on their unique needs. The intentional and strategic usage of sensory restoration and motor support based on the nature of the task showcases the potential of the NeuroSleeve system in providing adaptable assistance to individuals with hand impairments. Together, these findings demonstrate the feasibility of a personalized sensorimotor assistance strategy and provide early evidence of its efficacy, laying the groundwork for future larger-scale and pathology-stratified clinical trials.

The combination of electrical stimulation and exoskeletons will open up important possible follow-up investigation on the eventual rehabilitation implications thanks to the restoration of the sensory information and the consequent brain reactions (e.g., promotion of “positive” cortical plasticity) (*88*). Further research with larger cohorts will be crucial to confirm these effects, refine the SensoExo system’s effectiveness in real-world settings, and validate its application across a broader spectrum of hand impairments.

## MATERIALS AND METHODS

### Participant recruitment

This study enrolled a total of 14 subjects (48 ± 16 years, including 10 men) presenting with comparable hand sensory and motor impairments. Participants were recruited from different neurological conditions known to induce such impairments, including SCI (*n* = 6), stroke (*n* = 5), and brain injury (*n* = 3; comprising two cases of brain hemorrhage and one traumatic brain injury) (table S1). Ethical approval was obtained from both the ETH Zurich Ethical Committee (EK 2021-N-122) and the “Miroslav Zotović” rehabilitation clinic Ethical Committee (03-2105/1) to ensure compliance to good clinical practice. This study is registered on ClinicalTrials.gov under the identifier NCT05976087.

Before participation, all participants received written and verbal explanations of the study objectives, procedures, potential risks, and benefits. Participants were given sufficient time to ask questions and consider participation. Written informed consent was obtained from all participants before enrollment in the study. Participants were informed of their right to withdraw from the study at any time without consequences to their medical care.

Inclusion criteria required the presence of clinically assessed sensorimotor impairments of the hand (e.g., spatially focal unilateral stroke or SCI with American Spinal Injury Association (ASIA) impairement score C or D). Sensory function was evaluated using QST with a 10g Semmes-Weinstein monofilament, which assesses protective touch sensation. All included participants exhibited sensory deficits, ranging from complete loss of protective sensation (no perceived indentation of the monofilament across the hand) to altered tactile feedback (perception preserved only in limited locations). In addition, participants should retain sufficient proximal motor abilities, such as partial shoulder elevation (minimum of 45°) and/or elbow flexion-extension (minimum of 45°). This criterion was necessary to enable the participants to perform the functional tasks necessary for the study’s objectives.

### NeuroSleeve design

The NeuroSleeve was designed with a focus on both functionality and wearability, incorporating several key features: (i) modular design of TENS and FES delivery allowing personalized assistance to individual user needs; (ii) versatile design for interchangeable wear on either the right or left arm, accommodating user impairment and usability; (iii) consideration of anthropometric and anatomical variations between users, ensuring a comfortable and functional fit for a diverse range of individuals; (iv) intuitive donning and doffing with one hand, promoting independence for users with unilateral impairments; (v) seamless integration of electrodes and cabling into the sleeve fabric, enhancing device acceptance by users (*89*); (vi) the adoption of custom-sized electrodes made from conductive textile material addresses notable drawbacks associated with conventional sticky electrode pads. These limitations primarily revolve around size tailoring, contact stability, and conductivity, which can collectively contribute to uncomfortable and nonintuitive artificial sensations experienced by users.

### Pilot study in healthy participants drives NeuroSleeve design

The location and size of the stimulating electrodes are crucial to provide a somatotopic sensation in the hand (via median and ulnar TENS) and correct flexion and extension of the fingers (via finger muscle flexor and extensor FES). In this regard, we conducted a pilot study involving 10 healthy volunteers (five females and five males, age 23 ± 2 years) to investigate the effects of electrode location and size on sensory and motor nerve activation. For TENS, anode-cathode electrode pairs were placed in a radial configuration with respect to the wrist, inspired by the work of Pena and colleagues (*90*), where the anode was placed dorsally and the cathode ventrally in correspondence to the targeted sensory nerve. For FES, anode and cathode electrodes were both placed dorsally on the forearm in the case of ED stimulation and ventrally for FD stimulation. The optimal size and location of the electrodes were determined following a specifically defined optimization algorithm found in Supplementary Methods. Additional details on materials and design instead can be found in Supplementary Methods as well.

### Development of sensorized finger stalls

SensoExo aims to provide sensory feedback via TENS upon a touch event. While gloves may seem a straightforward solution to interface human fingertips with sensors and ultimately with the environment, they were found unsuitable for individuals with spasticity, commonly observed in stroke and SCI survivors, as they limit independent donning and doffing of the overall system. In addition, embedding sensors within a glove would make the system incompatible with many tight-fitting, glove-based soft hand exoskeletons (*91*). Notably, many existing exoskeletons, including the model used in this study, are already designed to be worn with gloves that are typically thicker than the proposed finger sleeves (*19*, *29*). Consequently, the use of finger sleeves does not further degrade or compromise residual sensory function beyond what is inherent to standard exoskeleton use.

Hence, we developed textile finger sleeves embedding capacitive force sensors over the thumb, index, and little fingertips which are worn by wrapping around the fingertip. This design offered customization in terms of adaptability to different finger sizes. Furthermore, the finger sleeves can be anchored to an exoskeleton or can be worn over it.

The capacitive force sensors used however suffered from two main limitations: high loss of functional range due to bending of their textile anchoring support and reduced sensing area with respect to the fingertip surface.

To address this issue, we 3D-printed a thin plate support using polylactic acid (PLA) to reduce preloading effects. The plate is placed at the bottom of the sensor (between the sensor and the fingertip), to prevent unwanted bending of the interface due to the fabric flexibility. The effects of the 3D-printed support were evaluated by looking at changes in the functional range loss during simple finger motion at no-load condition. In addition, we 3D-printed load concentrators using PLA and foam load transmitters and placed over the sensing area of the sensor to better account for nonperpendicular forces acting on the sensor. To evaluate the functional improvement of these components, we investigated the ability to detect grasp from simple sensors signal thresholding over 10 repetitions of three grasping types: palmar pinch, spherical grasp, and cylindrical grasp.

### SensoExo construction and closed-loop control

We developed two customized SensoExo variants. The RELab Tenoexo was enhanced (the Supplementary Materials) to incorporate inputs from the sensorized hand module and outputs to a portable stimulator, alongside a newly designed, compact, and fully portable 3D-printed backpack for housing these connections, actuators, and electronic components. The ARIES Lab’s CATCHGlove was designed and constructed de novo, from the hand module to the portable backpack (the Supplementary Materials). In addition, we created and integrated a dedicated control unit within the backpack to synchronize the inputs (from pressure and bending sensors in the hand module) and outputs (motor control signals), facilitating coordinated operation between the exoskeleton and the stimulation garment.

During object grasping with the SensoExo, the finger stalls act as the primary sensing unit, capturing both force exchange with the object and finger motion using bending sensors. These sensor values are collected and used by a wearable control unit to govern the stimulator. The stimulation is subsequently delivered through the NeuroSleeve’s TENS and FES modules (actuator units) to the user.

TENS stimulation is activated when the recorded force by sensors surpasses a defined threshold. Because of the flexibility of the finger stalls, the threshold is individually calibrated at the beginning of the session for each participant. The control unit then initiates stimulation specific toward the nerve innervating the finger experiencing the force. For the thumb and index finger, electrical stimulation targets the median nerve electrodes, while for the little finger, the ulnar nerve is stimulated. Stimulation intensity was controlled by linearly modulating the pulse width between participant-specific minimum and maximum values determined during calibration, corresponding to specific force levels. The lower pulse width bound was defined as the minimum value eliciting a clear somatotopic sensation, whereas the upper bound corresponded to the highest nonpainful stimulation level (fig. S6). Stimulation frequency was kept constant at 50 Hz, while stimulation amplitude was fixed after calibration and customized independently for each nerve and participant (*92*). Calibration was performed in two sequential steps. First, stimulation amplitude was calibrated using a continuous increasing ramp while keeping the pulse width fixed at 300 μs. Participants were instructed to report when the perceived somatotopic sensation reached an intensity of 5 on a 10-point scale (1 = barely noticeable, 10 = unbearable). The pulse width was calibrated by applying an increasing ramp while keeping the stimulation amplitude fixed at the previously determined value. Participants reported when the sensation intensity reached 2 out of 10 and 8 out of 10, which were defined as the minimum and maximum pulse width values, respectively, for subsequent modulation.

FES stimulation was synchronized with the exoskeleton assistance during both hand closure and opening, with charge delivery modulated according to the motor torque output of the exoskeleton. Pulse width was used to modulate stimulation intensity and was mapped to three discrete levels, linearly distributed between participant-specific minimum and maximum values, enabling graded finger flexion-extension with constant frequency at 30 Hz. During hand closure, stimulation was delivered exclusively to the FD muscles, whereas during hand opening, stimulation was applied only to the ED muscles.

FES calibration closely mirrored the TENS calibration procedure and was performed individually at the beginning of each session. First, stimulation amplitude was determined using a continuous increasing ramp while keeping the pulse width fixed at 300 μs. The amplitude was selected as the value that elicited finger flexion and extension spanning approximately half of the available RoM and was then fixed for the remainder of the session. Second, pulse width was calibrated by applying an increasing ramp while holding stimulation amplitude constant at the selected value. The minimum pulse width was defined as the value at which finger movement was first observed, whereas the maximum pulse width corresponded to the recruitment of the full finger RoM or the plateau at which further increases did not produce additional movement. These minimum and maximum pulse width values were associated with the lowest and highest stimulation levels, respectively, while the intermediate level was obtained via linear interpolation.

The SensoExo output torque levels can be regulated using the bending sensor embedded in the finger stalls (*93*, *94*). In cases where the subject lacks residual motor ability, push buttons are provided to manually open and close the exoskeleton (*19*). Similar to the bending sensors, three levels of force regulation are offered, adjustable by varying the duration of button presses.

### Sensory and motor impairment characterization

At the beginning of each session, all the participants went through a sensory and motor characterization. S02 and S05 opted to participate solely in the sensory assessment and did not proceed with the remaining part of the protocol.

For the motor characterization, the RoM was measured using a goniometer aligned with the MCP joint (*95*). The baseline was initially assessed by instructing the subject to open and close their impaired hand. The procedure is repeated three times. Subsequently, we evaluated the RoM improvement with the assistance of the exoskeleton, using the same technique. Participants with RoM impairment higher than 50% with respect to healthy ranges (−10° extension to 90° flexion) underwent further characterization while being assisted by SensoExo, which included FES targeting the fingers flexor and extensor muscles. The RoM for the different conditions was then compared, defining the restored RoM as the difference between the assisted and the nonassisted one.

For the sensory map, the subject was blindfolded, and a QST was conducted by applying a 10g Semmes-Weinstein monofilament to specific skin locations (*80*). The procedure was repeated twice. Subsequently, TENS was applied, and the participant indicated the location of the evoked artificial sensation on a hand model. The two maps were then digitized and compared. The intersection between the impaired sensory area and the artificially evoked sensory area was defined as the restored region.

### Functional tasks

Of the 14 enrolled participants, 8 participated in the GRT. During the GRT, participants were instructed to transport specific objects placed on one side of the table (corresponding to their impaired side) to the opposite side, overcoming an 8-cm high obstacle. To successfully complete each trial, the subjects needed to grasp the object with their impaired hand, lift it, place it in contact with the table, and release it. The trial was considered unsuccessful if the object’s contact was released before reaching the other side of the table after the first touch. Participants were instructed to use only their impaired hand. However, to ensure trial completion and maintain motivation, they were permitted to use their other hand if they felt uncomfortable or frustrated. Nevertheless, the trial was deemed unsuccessful if the unimpaired hand was used during the task.

The subject is requested to perform 30 repetitions of this task for four objects. Two bulky objects are as follows: a plastic cylinder of (15-cm height, 6.5-cm diameter, 120-g weight) and a plastic cube of (7.5-cm side length, 120-g weight). Two fragile objects are as follows: a virtual egg with a braking force at 200 gf and one at 100 gf (*96*). Each object was tested under three different conditions: no support, only exoskeleton, and SensoExo support. The order of conditions and object repetitions was randomized.

To accommodate each participant’s specific health and psychological condition, the duration and number of sessions were individually adjusted. Typically, the functional tasks were performed in sessions lasting around 30 min until completion. Because of the participants’ fragility, when elevated risk of drop out was assessed, priority was given to testing the no-support and SensoExo support conditions; order was kept randomized among the two conditions.

Throughout the trials, we recorded signals from all the different sources, including finger stall pressure, bending sensors, motor torque output, electrical stimulation, and control states. These data were then used to compute the exerted force, feedback utilization, and the system’s consumption among the different testing conditions.

### Statistical analysis

All data were exported and processed offline in MATLAB (R2022a, The MathWorks, Natick, USA) and R 4.3.2. The normality of data distributions was verified with a one-sample Kolmogorov-Smirnov test, using the MATLAB function “kstest.” The function returns a test decision for the null hypothesis that the given data come from a standard normal distribution, against the alternative that it does not come from such a distribution, using the one-sample Kolmogorov-Smirnov test. The result is 1 if the test rejects the null hypothesis at the 5% significance level or 0 otherwise. The RoM values were analyzed with a Friedman test, followed by Bonferroni post hoc correction for multiple groups comparison. The applied electrical charges were statistically compared through a Mann-Whitney-Wilcoxon test. Differences in the area size where sensory feedback is perceived, with and without stimulation, were compared using a Wilcoxon signed rank test. The GRT performances across conditions were initially compared using a pairwise chi-square test. Subsequently, a binomial generalized linear model was applied to correct the outcome, incorporating unique participant identifiers, testing conditions, and trial sequences as predictors. A Bonferroni post hoc correction for multiple comparisons was then conducted to characterize significant accuracy differences among testing conditions. On the presented boxplots, the central mark indicates the median, and the bottom and top edges of the box indicate the 25th and 75th percentiles, respectively. The whiskers extend to the most extreme data points not considered outliers, and the outliers are plotted individually using the “o” symbol. The level of significance was set at ***P < 0.001, **P < 0.01, and *P < 0.05. In the figure captions, we reported the statistical tests used for each analysis and its result, along with the number of repetitions (*n*) and *P* values for each experiment.

## Supplementary Materials

### The PDF file includes:

Supplementary Methods Figs. S1 to S6 Tables S1 and S2 Legends for movies S1 to S4 References
