# Supplementary material for: Merging neural stimulation and exoskeletons to enhance sensorimotor hand functions after brain or spinal cord injury
Source: Sci Adv. 2026 Jun 19;12(25):eady3144. doi: 10.1126/sciadv.ady3144 (PMC13281789; doi:10.1126/sciadv.ady3144)
Supplement: Supplementary file 1 — Supplementary Methods Figs. S1 to S6 Tables S1 and S2 Legends for movies S1 to S4 References [file sciadv.ady3144_sm.pdf]

Science Advances
AAAS

Supplementary Materials for

Merging neural stimulation and exoskeletons to enhance sensorimotor hand functions after brain or spinal cord injury

Andrea Cimolato *et al.*

Corresponding author: Stanisa Raspopovic, stanisa.raspopovic@meduniwien.ac.at

Sci. Adv. **12**, eady3144 (2026)
DOI: 10.1126/sciadv.ady3144

The PDF file includes:

Supplementary Methods
Figs. S1 to S6
Tables S1 and S2
Legends for movies S1 to S4
References

Other Supplementary Material for this manuscript includes the following:

Movies S1 to S4

Supplementary Methods:

Protocol for evaluating location of FES electrodes' effect on artificial finger motion.

For each participant, the best location for the FES electrodes was determined by solving the following optimization problem:

$$\max_{i,j} \text{rom\_fingers}_i + (1 - \text{rom\_wrist}_i)$$

$$\text{s.t. fingers\_rom}_i \leq 1;\ \ \forall i$$
$$\text{wrist\_rom}_i \leq 1;\ \forall i$$
$$i \in Positions$$
$$\text{fingers\_rom}_i \in \mathbb{R}_{\geq 0}, \forall i$$
$$wrist\_rom_i \in \mathbb{R}_{\geq 0}, \forall i$$

Where i represents all the tested electrode position; rom_fingers_i the percentage of metacarpophalangeal RoM elicited through FES from 0 to 1 for any combination of electrode placement; rom_wrist_i the percentage of flexion-extension RoM in wrist join elicited through FES from 0 to 1 for any combination of electrode placement. The solution to this problem provided the best placement of electrode that maximized the artificial finger motion while minimizing the undesired wrist flexion-extension. The same electrode size was maintained, corresponding to that of commercially available electrodes (Electrode Pads 50x50mm, TensCare Ltd, United Kingdom). No size optimization was performed as significant electrode size reduction would have caused uncomfortable sensations due to the high injected charge, and significant size increase would have impacted muscle selectivity. Indeed, elicitation of the ED and FD is only suboptimally precise due to their anatomical placement underneath the wrist flexor and extensor muscles, thus small changes in electrode size were deemed negligible.

Protocol for evaluating size and location of TENS electrodes' effect on artificial touch sensation.

For each participant, the best location and size of TENS electrodes were found by solving the following optimization problem:

$$\max_{i,j} \text{som}_{i,j} + \left(10 - \text{under\_el}_{i,j}\right)$$

$$\text{s.t. som}_{i,j} \leq 10;\ \ \forall i,j$$
$$\text{under\_el}_{i,j} \leq 10, \forall i,j$$
$$i \in [25, 21, 17, 13]$$
$$j \in Positions$$
$$\text{som}_{i,j} \in \mathbb{R}_{\geq 0}, \forall i,j$$
$$\text{under\_el}_{i,j} \in \mathbb{R}_{\geq 0}, \forall i,j$$

Where i represents the diameter (mm) of the tested electrodes; j represents all the tested electrode position; $\text{som}_{i,j}$ the intensity of the somatotopic sensation from 1 to 10, for any combination of electrode size and placement; $\text{under\_el}_{i,j}$ the intensity of the sensation under the electrode from 1 to 10, for any combination of electrode size and placement. The solution to this problem provided the best placement and size of electrode that maximized the artificial somatotopic sensation while minimizing the

sensation under the electrode. The process was repeated for both the median and the ulnar nerve stimulation.

Sleeve design features and materials.

The Neurosleeve support textile material was obtained from the customization of standard commercial compression sleeves (Compression arm sleeve performance, SupCare, Denmark). To improve wearability and facilitate easier electrodes adjustment, we crafted side openings on the elastic materials that could be closed and adjusted using elastic straps. Upon preliminary analysis of anthropometric variations between participants, it was concluded that a one-size-fits-all approach would not guarantee an adequate electrode adherence and a stable stimulation delivery even when employing compression garments. Hence, we developed three sleeve sizes (small, medium, large) with the same but scaled design. Furthermore, we accounted for anatomical variability in nerve location by embedding the textile electrodes into patches that could be moved within the garment during donning via attachment to Velcro guides sewed inside the sleeves. The Velcro guides were placed following the results of the electrode optimization study on healthy participants, and in a symmetrical fashion to allow interchangeable use on the right and left arm.

Following the determined optimal electrode size, TENS and FES electrodes made of conductive textile material (ElectroSkin GECKO, Nanoleq GmbH, Switzerland) were embedded into custom-made, so-called electrode patches made of fabric (Pompon GmbH, Switzerland). The shape of the patches was designed to match the respective NeuroSleeve modules, either TENS or FES, to which they attach with complementary Velcro guides. For both electrode patch types, we integrated the electrode pad on one side of the fabric via heat press, and we routed the electrical connection to come out on the other side of the fabric to a brass snap button. The snap button served as an electrical connection point between the electrode patch and custom-made textile cables. The textile stimulator-compatible cables were made using smart textile wire (Phantom Tape, Nanoleq GmbH, Switzerland), which we connected to the snap button's counterpart on one side, and to a 2mm diameter banana connector compatible with the stimulator's (RehaMove3, Hasomed GmbH, Germany) own output cables.

Concerning the sensor glove, the capacitive force sensors integrated into the finger-sleeves were selected based on two primary considerations: i) the functional range required to measure normal grasping forces (<500 gf) and ii) the need for a compact sensor design suitable for embedding on the surface of the fingertips. For the thumb and index finger, a larger sensor with a 9.53 mm diameter sensing area (FlexiForce A301, Tekscan Inc., United States) was chosen, while a force sensor with a 3.8 mm diameter sensing area (Flexi-Force A101, Tekscan, United States) was selected for the little finger-sleeve. In addition to the force sensors, a resistive flex sensor (Flex sensor, Spectra Symbol, United States) was strategically placed on the index finger, with anchor points on the dorsum of the hand and the index finger-sleeve. This configuration allowed accurate measure the metacarpophalangeal joint movements during grasping tasks.

TenoExo exoskeleton.

The RELab tenoexo is a fully wearable assistive hand exoskeleton developed to support grasp function in people with neurological hand impairment (*19*). It consists of a light-weight hand module (approx. 130 g) and an actuation module (approx. 560 g) containing electronics, battery, and two DC motors. The hand module is mounted on the back of a user's hand using glove with Velcro patches between glove and

exoskeleton, and straps rigidly fixing the exoskeleton at the wrist and metacarpus. The actuation module can be worn as a backpack or placed on a table or wheelchair. A Bowden cable-based remote actuation system transmits the power of the motors to the hand module (*72*), actuating finger flexion and extension via a three-layered guided spring mechanism (*97*). The fingers of the RELab tenoexo consist of rigid elements, mimicking the bones, and flexible spring elements, aligned along the joints of the human hand. A first bottom spring of fixed length rests on the back of the user's finger. A second spring placed at a small distance to the bottom spring is actively moved back and forth by the actuation system. Due to the created lever arm between these two spring layers, the mechanism bends along the spring elements when the second spring is moved. A third layer of springs on top of the second layer and the rigid elements constrain the bending motion biomimetically to the natural motion of a human hand.
Based on this mechanism, the RELab tenoexo actively supports flexion and extension of the index, middle, ring, and little finger combined, and the thumb separately. Additionally, the thumb can be manually moved to opposition. This combination of degrees of freedom allows the user to perform four grasp types (palmar pinch, medium wrap, parallel extension, and lateral pinch) relevant for daily grasping tasks.
Previous studies have shown that the RELab tenoexo can generate an immediate benefit in functional performance in people with hand impairment after a spinal cord injury (*98*) and bimanual hand performance in children minimal hand function due to neurological impairments (*99*).

CATCHglove exoskeleton.
The development of the glove, known as the Custom Assistive Tendon-driven Clutchable Hand (CATCHglove), has the primary goal to faithfully replicate hand functions by assisting all five fingers. To simplify the complexity of the mechanism, we chose a one-to-many strategy that exploits the redundancy and synergies inherent in hand grasping. This enabled us to actuate the entire hand using three motors that actively aid in flexion, while a passive leaf spring mechanism (Hoffman Group, Knoxville, TN, USA) facilitates the extension.
The base of the CATCHglove is a commercially available golf glove (Dawn Patrol, Callaway Golf, Carlsbad, CA, US) that has been ad-hoc modified to improve the wearability for people affected by muscular diseases splitting the textile component into three parts that can be linked independently as depicted in (Supplementary Figure 1).
We utilized a sewn-on guiding system with Teflon tubes held in place by textile parts and 3D printed components, guiding the tendons along the palm, and ensuring a well-distributed pulling force. The tendons were directed around the fingertips of the assisted fingers in three groups (thumb, index & middle finger, and ring & pinkie finger) to promote hand synergies as extensively described by In et al (*100*). To establish a secure connection between the wrist and the actuation unit, we employed a Bowden cable system (Shimano, SLR, Ø 4 mm, Sakai, Ōsaka, Japan) that could be magnetically secured with a clutchable customized 3D-printed system (*101*).
To monitor the overall bending angle during flexion and provide position feedback, we integrated three flex sensors (Bend Labs, Salt Lake City, UT, USA) at the thumb, index finger level, and ring finger. These sensors communicate the finger kinematic information to the controller architecture, as described in Missiroli et al. (*102*). An Arduino MKR 1010 WiFi (Arduino, Ivrea, Italy), serving as the real-time control unit, running at 100 Hz, executes the real-time controller implemented in a MATLAB/Simulink application (MathWorks, Natick, Massachusetts MA, USA) and sends motor commands via CAN-bus to the actuation stage.

The actuation stage, responsible for facilitating finger flexion, consists of three flat brushless motors (T-Motor, AK60-6, 24V, 6:1 planetary gear-head reduction, Cube Mars actuator, T-MOTOR, Nanchang, Jiangxi, China). Each motor drives a pulley (Ø 35mm), around which an artificial tendon is wound to facilitate finger movement.

Supplementary Figures:

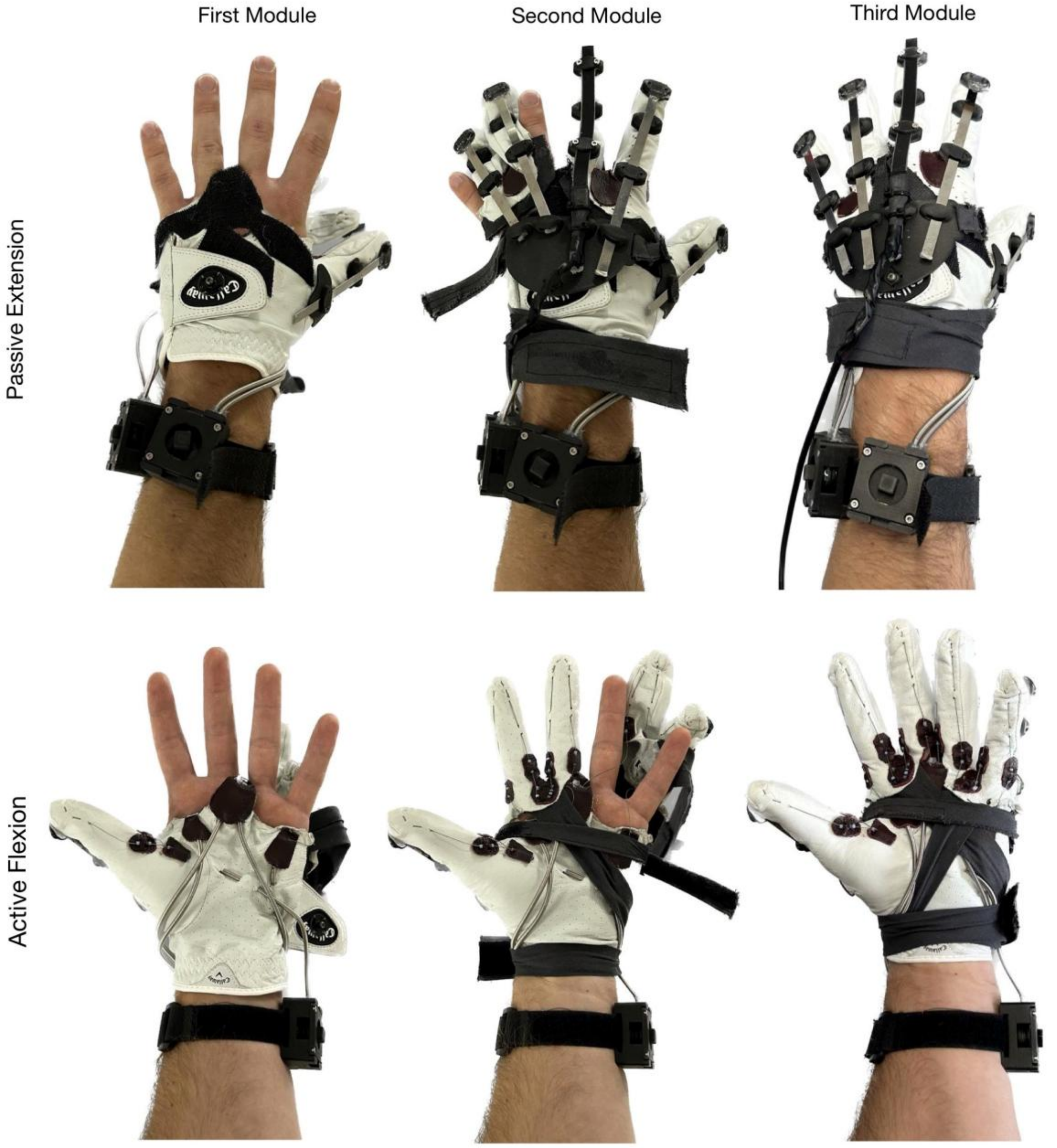

Fig. S1. Design of the CATCHglove. From left to right the three modular parts of the glove can be worn independently by the subject. Top: the active side that actuates the finger flexion via three independent tendon-driven actuators. Bottom: passive opening via harmonic steel and flex sensors to detect the user's kinematics.

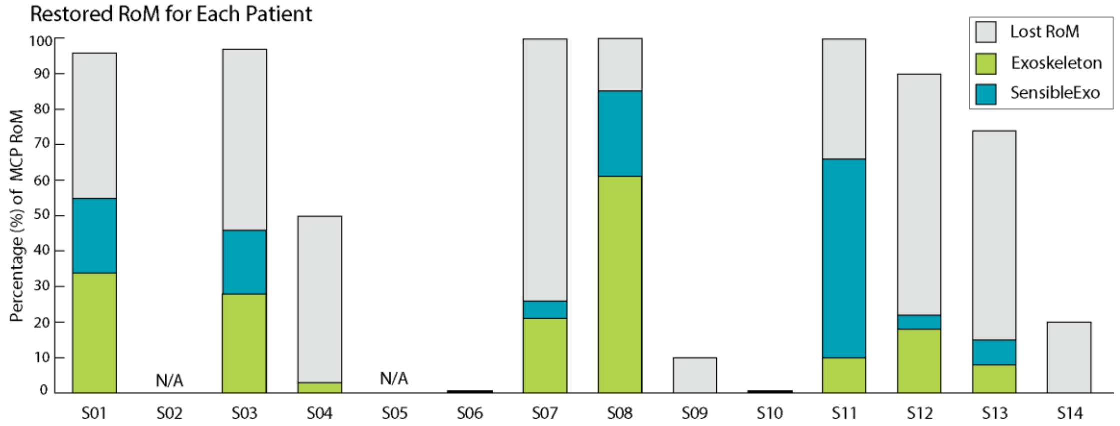

Fig. S2. Lost and restored RoM in all patients. The bar plot shows, in light grey, the amount of lost RoM for each participant, on top of that (in blue) the amount of the lost RoM that was restored using the SensoExo, and at last on top of that (in green) the amount of the lost RoM that was restored using only soft exoskeleton support.

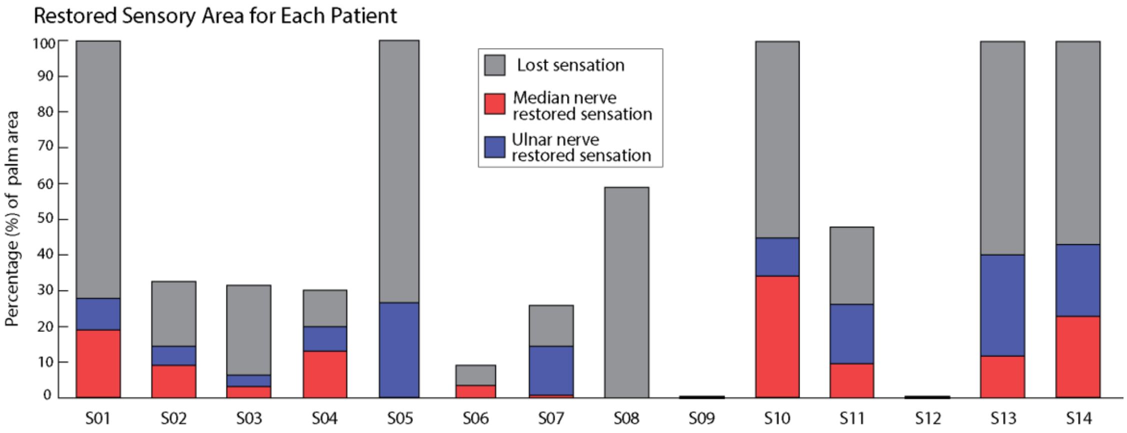

Fig. S3. Lost and restored sensory area in all patients. The bar plot shows in grey the amount of lost sensory palmar area for each participant, and how much of that lost percentage has been recovered combining median TENS (red) and ulnar TENS (blue).

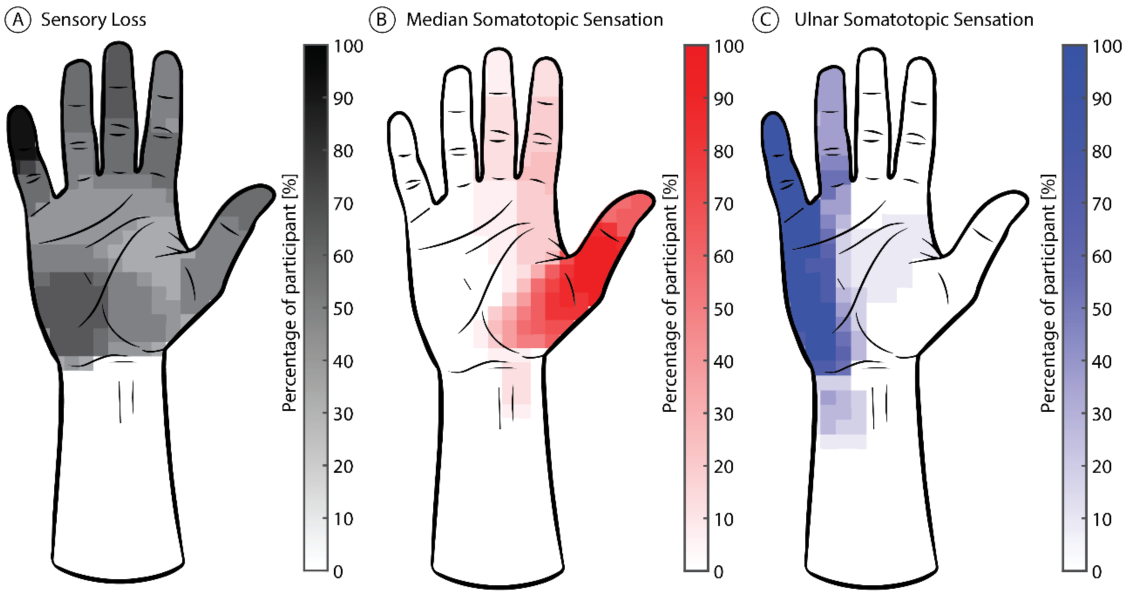

Fig. S4. Sensory maps across the entire participant cohort. Heatmaps illustrate the percentage of patients reporting sensory characterization in specific hand regions. A) Areas insensitive to quantitative sensory testing (QST); B) Regions exhibiting somatotopic tactile sensation evoked by median nerve TENS; C) Regions exhibiting somatotopic tactile sensation evoked by ulnar nerve TENS. Color intensity indicates the proportion of participants (%) reporting sensory changes at each location.

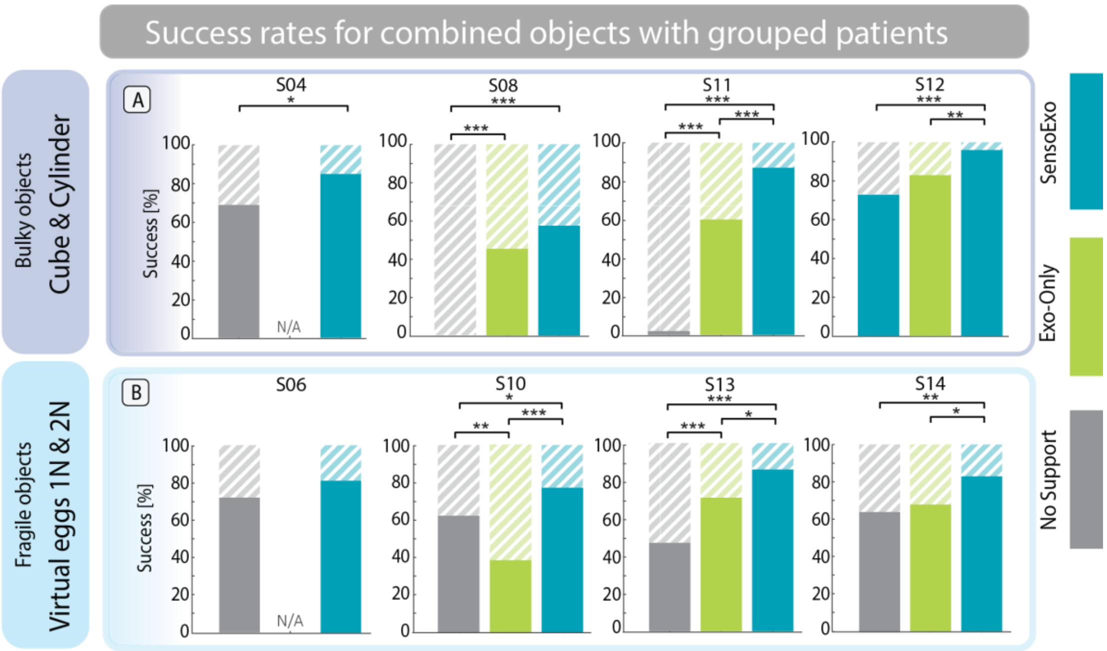

Fig. S5. Functional benefits of using SensoExo during functional tasks for all objects. Bar plot illustrating the performance improvement in grasp and release tasks under three conditions: no support, only exoskeleton support, and SensoExo support. Performance of the subjects is grouped for all the bulky objects on top, and for all the fragile objects, on the bottom. In A, patients with higher sensory loss exhibit significant enhancement in handling fragile objects using SensoExo. In B, patients with higher motor loss show significant improvement in manipulating large objects (cylinder and cube) when using SensoExo. Chi-Square test was used to first assess the significance of test condition. In the figures are reported the results for the pairwise comparison. P-values legend: * p<0.05, ** p<0.01, *** p<0.001.

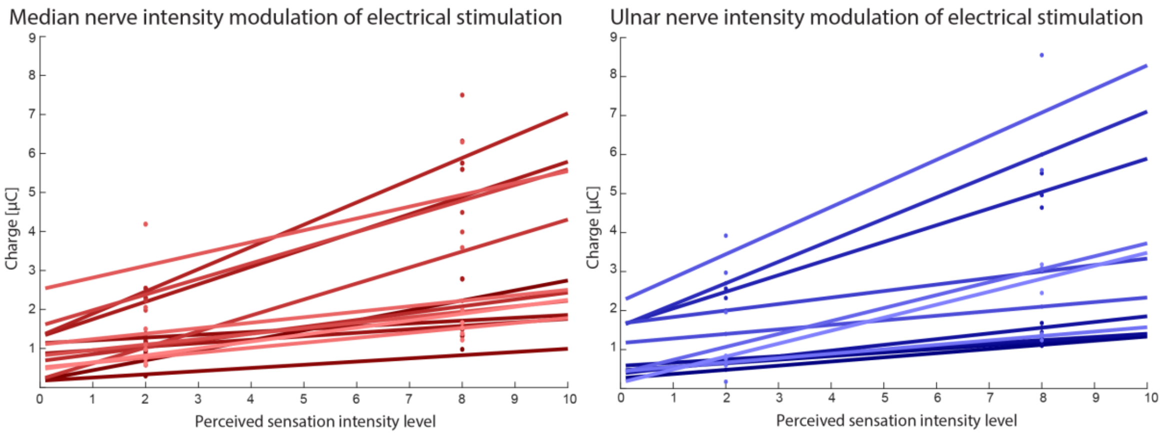

Fig. S6. Intensity modulation curves of TENS intensity across all patients. Each patient's data is shown in varying shades of blue & red. The scatter plots represent the delivered stimulation charge in relation to the perceived sensation intensity (patients were asked to report only when the sensation reached levels 2 and 8 out of 10). For each patient, a linear fit is included, depicting the characterization curve of the injected charge with respect to the reported intensity. Three repetitions were recorded per intensity level per patient. The left panel shows TENS characterization for the median nerve, while the right panel presents data for the ulnar nerve.

Supplementary Tables:

Subject ID	Age (years)	Condition	*Sensory function assessment (Loss of sensation according to QST)*	*Motor function assessment (FMA-hand ≤ 12 or AIS-Upper Extremities ≤ 20)*	*NeuroSleeve Modules*		*Control*	Soft Exoskeleton	Sensory Characterization	Motor Characterization	Grasp and Release Tasks	*Cause of incomplete evaluation*
					TENS	*FES*						
S01	49	SCI (ASIA A)	*YES*	*AIS-Upper Extremities 4*	*YES*	*YES*	*Buttons*	TenoExo	YES	YES	NO	*Health complication*
S02	38	SCI (ASIA A)	*YES*	*AIS-Upper Extremities 15*	*YES*	*NO*	*/*	TenoExo	YES	NO	NO	*Health complication*
S03	51	Traumatic brain injury	*YES*	*FMA-hand 2*	*YES*	*YES*	*Buttons*	TenoExo	YES	YES	NO	*Time constraint*
S04	72	Stroke	*YES*	*FMA-hand 12*	*YES*	*NO*	*Flex sensor*	TenoExo	YES	YES	YES	*N/A*
S05	64	Stroke	*YES*	*FMA-hand 3*	*YES*	*NO*	*/*	TenoExo	YES	NO	NO	*Time constraint*
S06	77	Brain hemorrhage	*YES*	*FMA-hand 12*	*YES*	*NO*	*Flex sensor*	CATCHGlove	YES	YES	YES	*N/A*
S07	32	SCI (ASIA A)	*YES*	*AIS-Upper Extremities 13*	*YES*	*YES*	*Buttons*	CATCHGlove	YES	YES	NO	*Health complication*
S08	29	SCI (ASIA A)	*YES*	*AIS-Upper Extremities 3*	*YES*	*YES*	*Buttons*	CATCHGlove	YES	YES	YES	*N/A*
S09	58	Stroke	*YES*	*FMA-hand 7*	*YES*	*NO*	*Flex sensor*	CATCHGlove	YES	YES	NO	*Health complication*
S10	43	Traumatic brain injury	*YES*	*FMA-hand 12*	*YES*	*NO*	*Flex sensor*	TenoExo	YES	YES	YES	*N/A*
S11	50	Stroke	*YES*	*FMA-hand 2*	*YES*	*YES*	*Buttons*	TenoExo	YES	YES	YES	*N/A*
S12	35	SCI (ASIA A)	*YES*	*AIS-Upper Extremities 13*	*YES*	*YES*	*Buttons*	TenoExo	YES	YES	YES	*N/A*
S13	20	SCI (ASIA A)	*YES*	*AIS-Upper Extremities 3*	*YES*	*YES*	*Buttons*	TenoExo	YES	YES	YES	*N/A*
S14	48	Stroke	*YES*	*FMA-hand 8*	*YES*	*NO*	*Flex sensor*	TenoExo	YES	YES	YES	*N/A*

Table S1. Participants summary table. Clinical condition, age, and motor and sensory function assessments relative to the inclusion criteria for each participant. For each participant, the type of support provided, tests performed, and reasons for early termination of the protocol (if applicable) are also reported.

Participant ID	Median TENS			Ulnar TENS			Finger Flex. FES			Finger Ext. FES		
	Amp [mA]	PW Min [μs]	PW Max [μs]	Amp [mA]	PW Min [μs]	PW Max [μs]	Amp [mA]	PW Min [μs]	PW Max [μs]	Amp [mA]	PW Min [μs]	PW Max [μs]
S01	9	80	250	4	120	280	18	140	360	14	150	220
S02	3	120	270	5	140	220						
S03	5	240	350	4	220	320	17	120	430	19	200	600
S04	4	260	400	4	170	360						
S05	7	300	700	6	270	430						
S06	8	320	720	8	320	580						
S07	7	150	300	15	180	400	16	350	450	19	350	400
S08	7	150	500	9	130	450	6	100	200	17	100	350
S09	12	200	400	10	200	300						
S10	7	160	280	7	200	300						
S11	9	230	400	9	330	950	20	100	250	35	200	370
S12	8	160	280	7	280	440	17	250	640	13	220	400
S13	4	150	350	4	160	350	14	230	290	15	290	640
S14	7	130	280	5	160	490						

Table S2. Participants stimulation parameters for TENS and FES summary table. Participant-specific stimulation parameters for median and ulnar transcutaneous electrical nerve stimulation and functional electrical stimulation of finger flexor and extensor muscles. The table reports stimulation amplitude and minimum–maximum pulse width ranges; pulse frequency was fixed at 50 Hz for all participants. Empty entries indicate stimulation modalities not applied based on individual impairment profiles.

Supplementary Movies:

Movie S1. SensoExo overview on architecture, control and working principle.

Movie S2. Example of a functional task trial during the unassisted condition, employing the exoskeleton alone and the SensoExo. The task represents a common daily task, such as drinking from a bottle.

Movie S3. Example of Grasp and Release Test with bulky objects. The video demonstrates the participant facing difficulty in task completion due to elevated hand spasticity. The use of SensoExo significantly enhanced the participant's finger range of motion and grip force, enabling successful task execution.

Movie S4. Example of Grasp and Release Test with fragile objects. The video showcases the participant unable to accomplish the task due to missing sensory feedback from his palm of the hand. The use of SensoExo boosts his ability to fine control the exoskeleton device in order to accomplish the task with higher accuracy.

REFERENCES

1. V. L. Feigin, M. Brainin, B. Norrving, S. Martins, R. L. Sacco, W. Hacke, M. Fisher, J. Pandian, P. Lindsay, World Stroke Organization (WSO): Global stroke fact sheet 2022. *Int. J. Stroke* **17**, 18–29 (2022).

2. National Spinal Cord Injury Statistical Center, *Spinal Cord Injury Facts and Figures at a Glance* (University of Alabama at Birmingham, 2020).

3. S. Hunter, P. Crome, Hand function and stroke. *Rev. Clin. Gerontol.* **12**, 68–81 (2002).

4. R. M. Dannenbaum, R. W. Dykes, Sensory loss in the hand after sensory stroke: Therapeutic rationale. *Arch. Phys. Med. Rehabil.* **69**, 833–839 (1988).

5. L. M. Carey, T. A. Matyas, L. E. Oke, Sensory loss in stroke patients: Effective training of tactile and proprioceptive discrimination. *Arch. Phys. Med. Rehabil.* **74**, 602–611 (1993).

6. N. B. Finnerup, I. L. Johannesen, A. Fuglsang-Frederiksen, F. W. Bach, T. S. Jensen, Sensory function in spinal cord injury patients with and without central pain. *Brain* **126**, 57–70 (2003).

7. S. T. Fujimoto, L. Longhi, K. E. Saatman, T. K. McIntosh, Motor and cognitive function evaluation following experimental traumatic brain injury. *Neurosci. Biobehav. Rev.* **28**, 365–378 (2004).

8. R. M. Dannenbaum, L. A. Jones, The assessment and treatment of patients who have sensory loss following cortical lesions. *J. Hand Ther.* **6**, 130–138 (1993).

9. G. Kwakkel, B. J. Kollen, J. van der Grond, A. J. H. Prevo, Probability of regaining dexterity in the flaccid upper limb. *Stroke* **34**, 2181–2186 (2003).

10. Y. Yun, Y. Na, P. Esmatloo, S. Dancausse, A. Serrato, C. A. Merring, P. Agarwal, A. D. Deshpande, Improvement of hand functions of spinal cord injury patients with electromyography-driven hand exoskeleton: A feasibility study. *Wearable Technol.* **1**, e8 (2020).

11. H. S. Jørgensen, H. Nakayama, P. M. Pedersen, L. Kammersgaard, H. O. Raaschou, T. S. Olsen, Epidemiology of stroke-related disability: The copenhagen stroke study. *Clin. Geriatr. Med.* **15**, 785–800 (1999).

12. D. T. Wade, Measuring arm impairment and disability after stroke. *Int. Disabil. Stud.* **11**, 89–92 (1989).

13. M. J. Fuhrer, D. H. Rintala, K. A. Hart, R. Clearman, M. E. Young, Relationship of life satisfaction to impairment, disability, and handicap among persons with spinal cord injury living in the community. *Arch. Phys. Med. Rehabil.* **73**, 552–557 (1992).

14. P. Fougeyrollas, L. Noreau, Long-term consequences of spinal cord injury on social participation: The occurrence of handicap situations. *Disabil. Rehabil.* **22**, 170–180 (2000).

15. F. Aggogeri, T. Mikolajczyk, J. O'Kane, Robotics for rehabilitation of hand movement in stroke survivors. *Adv. Mech. Eng.* **11**, 168781401984192 (2019).

16. J. O'Neill, T. A. Dyson-Hudson, Employment after spinal cord injury. *Curr. Phys. Med. Rehabil. Rep.* **8**, 141–148 (2020).

17. A. Beedie, P. Kennedy, Quality of social support predicts hopelessness and depression post spinal cord injury. *J. Clin. Psychol. Med. Settings* **9**, 227–234 (2002).

18. R. Müller, C. Peter, A. Cieza, M. W. Post, C. M. Van Leeuwen, C. S. Werner, S. Geyh, O. Dériaz, M. Baumberger, H. P. Gmünder, A. Curt, M. Schubert, K. Hug, M. Hund-Georgiadis, H. G. Koch, U. Styger, H. Landolt, H. Koch, M. Brach, G. Stucki, M. Brinkhof, C. Thyrian, Social skills: A resource for more social support, lower depression levels, higher quality of life, and participation in individuals with spinal cord injury? *Arch. Phys. Med. Rehabil.* **96**, 447–455 (2015).

19. T. Bützer, O. Lambercy, J. Arata, R. Gassert, Fully wearable actuated soft exoskeleton for grasping assistance in everyday activities. *Soft Robot.* **8**, 128–143 (2021).

20. K. S. Beekhuizen, New perspectives on improving upper extremity function after spinal cord injury. *J. Neurol. Phys. Ther.* **29**, 157–162 (2005).

21. S. Barreca, S. L. Wolf, S. Fasoli, R. Bohannon, Treatment interventions for the paretic upper limb of stroke survivors: A critical review. *Neurorehabil. Neural Repair* **17**, 220–226 (2003).

22. J. H. van der Lee, I. A. Snels, H. Beckerman, G. J. Lankhorst, R. C. Wagenaar, L. M. Bouter, Exercise therapy for arm function in stroke patients: A systematic review of randomized controlled trials. *Clin. Rehabil.* **15**, 20–31 (2001).

23. V. Dietz, L. Marchal-Crespo, D. Reinkensmeyer, "Recovery of sensorimotor functions after stroke and SCI: Neurophysiological basis of rehabilitation technology," in *Neurorehabilitation Technology*, D. J. Reinkensmeyer, L. Marchal-Crespo, V. Dietz, Eds. (Springer International Publishing, 2022), pp. 41–53.

24. O. Lambercy, L. Dovat, H. Yun, S. K. Wee, C. W. Kuah, K. S. Chua, R. Gassert, T. E. Milner, C. L. Teo, E. Burdet, Effects of a robot-assisted training of grasp and pronation/supination in chronic stroke: A pilot study. *J. NeuroEngineering Rehabil.* **8**, 63 (2011).

25. S. S. Groothuis, S. Stramigioli, R. Carloni, Lending a helping hand: Toward novel assistive robotic arms. *IEEE Robot. Autom. Mag.* **20**, 20–29 (2013).

26. H. S. Nam, H. G. Seo, J.-H. Leigh, Y. J. Kim, S. Kim, M. S. Bang, External robotic arm vs. upper limb exoskeleton: What do potential users need? *Appl. Sci.* **9**, 2471 (2019).

27. J. M. Catalán, E. Trigili, M. Nann, A. Blanco-Ivorra, C. Lauretti, F. Cordella, E. Ivorra, E. Armstrong, S. Crea, M. Alcañiz, L. Zollo, S. R. Soekadar, N. Vitiello, N. García-Aracil, Hybrid brain/neural interface and autonomous vision-guided whole-arm exoskeleton control to perform activities of daily living (ADLs). *J. Neuroeng. Rehabil.* **20**, 61 (2023).

28. V. Klamroth-Marganska, V. M. Miller, Eds., "Stroke rehabilitation: Therapy robots and assistive devices," in *Sex-Specific Analysis of Cardiovascular Function* (Springer International Publishing, 2018), vol. 1065 of *Advances in Experimental Medicine and Biology*, pp. 579–587.

29. T. Shahid, D. Gouwanda, S. G. Nurzaman, A. A. Gopalai, Moving toward soft robotics: A decade review of the design of hand exoskeletons. *Biomimetics* **3**, 17 (2018).

30. H. K. Yap, J. H. Lim, F. Nasrallah, J. C. H. Goh, R. C. H. Yeow, “A soft exoskeleton for hand assistive and rehabilitation application using pneumatic actuators with variable stiffness,” in *2015 IEEE International Conference on Robotics and Automation* (*ICRA*) (IEEE, 2015), pp. 4967–4972.

31. R. A. Bos, C. J. W. Haarman, T. Stortelder, K. Nizamis, J. L. Herder, A. H. A. Stienen, D. H. Plettenburg, A structured overview of trends and technologies used in dynamic hand orthoses. *J. Neuroeng. Rehabil.* **13**, 62 (2016).

32. A. F. Pérez Vidal, J. Y. Rumbo Morales, G. Ortiz Torres, F. de Jesus Sorcia Vázquez, A. Cruz Rojas, J. A. Brizuela Mendoza, J. C. Rodríguez Cerda, Soft exoskeletons: Development, requirements, and challenges of the last decade. *Actuators* **10**, 166 (2021).

33. N. Li, T. Yang, P. Yu, J. Chang, L. Zhao, X. Zhao, I. H. Elhajj, N. Xi, L. Liu, Bio-inspired upper limb soft exoskeleton to reduce stroke-induced complications. *Bioinspir. Biomim.* **13**, 066001 (2018).

34. N. Lotti, F. Missiroli, E. Galofaro, E. Tricomi, D. Di Domenico, M. Semprini, M. Casadio, G. Brichetto, L. De Michieli, A. Tacchino, L. Masia, Soft robotics to enhance upper limb endurance in individuals with multiple sclerosis. *Soft Robot.* **11**, 243–255 (2024).

35. L. Cappello, J. T. Meyer, K. C. Galloway, J. D. Peisner, R. Granberry, D. A. Wagner, S. Engelhardt, S. Paganoni, C. J. Walsh, Assisting hand function after spinal cord injury with a fabric-based soft robotic glove. *J. Neuroeng. Rehabil.* **15**, 59 (2018).

36. L. Morris, R. S. Diteesawat, N. Rahman, A. Turton, M. Cramp, J. Rossiter, The-state-of-the-art of soft robotics to assist mobility: A review of physiotherapist and patient identified limitations of current lower-limb exoskeletons and the potential soft-robotic solutions. *J. Neuroeng. Rehabil.* **20**, 18 (2023).

37. A. J. Veale, S. Q. Xie, Towards compliant and wearable robotic orthoses: A review of current and emerging actuator technologies. *Med. Eng. Phys.* **38**, 317–325 (2016).

38. M. Decker, Y. Kim, "A hand exoskeleton device for robot assisted sensory-motor training after stroke," in *2017 IEEE World Haptics Conference* (*WHC*) (IEEE, 2017), pp. 436–441.

39. C. Marquez-Chin, M. R. Popovic, Functional electrical stimulation therapy for restoration of motor function after spinal cord injury and stroke: A review. *Biomed. Eng. Online* **19**, 1–25 (2020).

40. I. W. Baumgart, M. J. Darrow, N. J. Tacca, C. F. Dunlap, S. C. Colachis, A. Kamath, B. R. Schlink, P. T. Putnam, J. Branch, D. A. Friedenberg, L. R. Wengerd, E. C. Meyers, Restoration of upper-extremity function after task-oriented, intention-driven functional electrical stimulation therapy using a wearable sleeve in adults with chronic stroke: A case series. medRxiv 24301486 [Preprint] (2024). https://doi.org/10.1101/2024.01.18.24301486.

41. P. H. Peckham, J. S. Knutson, Functional electrical stimulation for neuromuscular applications. *Annu. Rev. Biomed. Eng.* **7**, 327–360 (2005).

42. K. Yang, C. Freeman, R. Torah, S. Beeby, J. Tudor, Screen printed fabric electrode array for wearable functional electrical stimulation. *Sens. Actuators A Phys.* **213**, 108–115 (2014).

43. N. M. Malešević, L. Z. P. Maneski, V. Ilić, N. Jorgovanović, G. Bijelić, T. Keller, D. B. Popović, A multi-pad electrode based functional electrical stimulation system for restoration of grasp. *J. Neuroeng. Rehabil.* **9**, 66 (2012).

44. J. Ciancibello, K. King, M. A. Meghrazi, S. Padmanaban, T. Levy, R. Ramdeo, M. Straka, C. Bouton, Closed-loop neuromuscular electrical stimulation using feedforward-feedback control and textile electrodes to regulate grasp force in quadriplegia. *Bioelectron. Med.* **5**, 19 (2019).

45. M. Liu, T. Ward, O. Keim, Y. Yin, P. Taylor, J. Tudor, K. Yang, Design and test of E-textiles for stroke rehabilitation. *Eng. Proc.* **30**, 16 (2023).

46. R. Bennett, C. McDonnell, D. Tyler, J. Wood, "A wearable FES compression garment," in *International Conference on the Challenges*, *Opportunities*, *Innovations and Applications in Electronic Textiles* (MDPI, 2019), p. 17.

47. K. Yang, K. Meadmore, C. Freeman, N. Grabham, A.-M. Hughes, Y. Wei, R. Torah, M. Glanc-Gostkiewicz, S. Beeby, J. Tudor, Development of user-friendly wearable electronic textiles for healthcare applications. *Sensors* **18**, 2410 (2018).

48. A. Prochazka, M. Gauthier, M. Wieler, Z. Kenwell, The bionic glove: An electrical stimulator garment that provides controlled grasp and hand opening in quadriplegia. *Arch. Phys. Med. Rehabil.* **78**, 608–614 (1997).

49. M. A. Khan, M. Saibene, R. Das, I. Brunner, S. Puthusserypady, Emergence of flexible technology in developing advanced systems for post-stroke rehabilitation: A comprehensive review. *J. Neural Eng.* **18**, 061003 (2021).

50. M. Gobbo, N. A. Maffiuletti, C. Orizio, M. A. Minetto, Muscle motor point identification is essential for optimizing neuromuscular electrical stimulation use. *J. Neuroeng. Rehabil.* **11**, 17 (2014).

51. S. Yang, R. Li, H. Li, K. Xu, Y. Shi, Q. Wang, T. Yang, X. Sun, Exploring the use of brain-computer interfaces in stroke neurorehabilitation. *Biomed. Res. Int.* **2021**, e9967348 (2021).

52. P. Ragert, T. Kalisch, B. Bliem, S. Franzkowiak, H. R. Dinse, Differential effects of tactile high- and low-frequency stimulation on tactile discrimination in human subjects. *BMC Neurosci.* **9**, 9 (2008).

53. J. C. Kattenstroth, T. Kalisch, M. Sczesny-Kaiser, W. Greulich, M. Tegenthoff, H. R. Dinse, Daily repetitive sensory stimulation of the paretic hand for the treatment of sensorimotor deficits in patients with subacute stroke: Reset, a randomized, sham-controlled trial. *BMC Neurol.* **18**, 2 (2018).

54. J.-C. Metzger, O. Lambercy, A. Califfi, F. M. Conti, R. Gassert, Neurocognitive robot-assisted therapy of hand function. *IEEE Trans. Haptics* **7**, 140–149 (2014).

55. A. Crema, M. Bassolino, E. Guanziroli, M. Colombo, O. Blanke, A. Serino, S. Micera, F. Molteni, Neuromuscular electrical stimulation restores upper limb sensory-motor functions and body representations in chronic stroke survivors. *Med* **3**, 58–74.e10 (2022).

56. D. G. Kamper, “Restoration of hand function in stroke and spinal cord injury,” in *Neurorehabilitation Technology*, D. J. Reinkensmeyer, V. Dietz, Eds. (Springer International Publishing, 2016), pp. 311–331.

57. Y. Laufer, M. Elboim-Gabyzon, Does sensory transcutaneous electrical stimulation enhance motor recovery following a stroke? A systematic review. *Neurorehabil. Neural Repair* **25**, 799–809 (2011).

58. C. Ethier, E. R. Oby, M. J. Bauman, L. E. Miller, Restoration of grasp following paralysis through brain-controlled stimulation of muscles. *Nature* **485**, 368–371 (2012).

59. C. T. Moritz, S. I. Perlmutter, E. E. Fetz, Direct control of paralysed muscles by cortical neurons. *Nature* **456**, 639–642 (2008).

60. M. Badi, S. Wurth, I. Scarpato, E. Roussinova, E. Losanno, A. Bogaard, M. Delacombaz, S. Borgognon, P. Čvančara, F. Fallegger, D. K. Su, E. Schmidlin, G. Courtine, J. Bloch, S. P. Lacour, T. Stieglitz, E. M. Rouiller, M. Capogrosso, S. Micera, Intrafascicular peripheral nerve stimulation produces fine functional hand movements in primates. *Sci. Transl. Med.* **13**, eabg6463 (2021).

61. A. B. Ajiboye, F. R. Willett, D. R. Young, W. D. Memberg, B. A. Murphy, J. P. Miller, B. L. Walter, J. A. Sweet, H. A. Hoyen, M. W. Keith, P. H. Peckham, J. D. Simeral, J. P. Donoghue, L. R. Hochberg, R. F. Kirsch, Restoration of reaching and grasping movements through brain-controlled muscle stimulation in a person with tetraplegia: A proof-of-concept demonstration. *Lancet* **389**, 1821–1830 (2017).

62. E. Z. Herring, E. L. Graczyk, W. D. Memberg, R. Adams, G. Fernandez Baca-Vaca, B. C. Hutchison, J. T. Krall, B. J. Alexander, E. C. Conlan, K. E. Alfaro, P. Bhat, A. B. Ketting-Olivier, C. A. Haddix, D. M. Taylor, D. J. Tyler, J. A. Sweet, R. F. Kirsch, A. B. Ajiboye, J. P. Miller, Reconnecting the hand and arm to the brain: Efficacy of neural interfaces for sensorimotor restoration after tetraplegia. *Neurosurgery* **94**, 864–874 (2024).

63. A. S. Gorgey, Robotic exoskeletons: The current pros and cons. *World J. Orthop.* **9**, 112–119 (2018).

64. B. Moineau, M. Myers, S. Ali, M. R. Popovic, S. L. Hitzig, End-user and clinician perspectives on the viability of wearable functional electrical stimulation garments after stroke and spinal cord injury. *Assist. Technol.* **34**, 453–462 (2022).

65. P. Slade, M. J. Kochenderfer, S. L. Delp, S. H. Collins, Personalizing exoskeleton assistance while walking in the real world. *Nature* **610**, 277–282 (2022).

66. M. M. Adams, A. L. Hicks, Spasticity after spinal cord injury. *Spinal Cord* **43**, 577–586 (2005).

67. G. J. Snoek, M. J. IJzerman, H. J. Hermens, D. Maxwell, F. Biering-Sorensen, Survey of the needs of patients with spinal cord injury: Impact and priority for improvement in hand function in tetraplegics. *Spinal Cord* **42**, 526–532 (2004).

68. M. Farzad, A. Asgari, F. Dashab, F. Layeghi, M. Karimlou, S. A. Hosseini, M. Rassafiani, Does disability correlate with impairment after hand injury? *Clin. Orthop. Relat. Res.* **473**, 3470–3476 (2015).

69. S. Kus, L. A. Van De Ven-Stevens, M. Coenen, S. Berno, B. Kollerits, A. Cieza, What is our knowledge of functioning and disability in hand conditions based on? *Arch. Phys. Med. Rehabil.* **92**, 1326–1332 (2011).

70. D. Weber, A. Matsiko, Assistive robotics should seamlessly integrate humans and robots. *Sci. Robot.* **8**, eadl0014 (2023).

71. J. T. Meyer, J. Dittli, A. Stutz, O. Lambercy, R. Gassert, "A method to evaluate and improve the usability of a robotic hand orthosis from the caregiver perspective," in *2020 8th IEEE RAS/EMBS International Conference for Biomedical Robotics and Biomechatronics* (*BioRob*) (IEEE, 2020), pp. 605–610.

72. J. Dittli, U. A. T. Hofmann, T. Bützer, G. Smit, O. Lambercy, R. Gassert, Remote actuation systems for fully wearable assistive devices: Requirements, selection, and optimization for out-of-the-lab application of a hand exoskeleton. *Front. Robot. AI* **7**, 596185 (2021).

73. V. Bucciarelli, N. Gozzi, N. Katic, G. Aiello, M. Razzoli, G. Valle, S. Raspopovic, Multiparametric non-linear TENS modulation to integrate intuitive sensory feedback. *J. Neural Eng.* **20**, 036026 (2023).

74. N. Gozzi, L. Chee, I. Odermatt, S. Kikkert, G. Preatoni, G. Valle, N. Pfender, F. Beuschlein, N. Wenderoth, C. Zipser, S. Raspopovic, Wearable neuroprosthesis improves mobility and reduces pain in neuropathic participants. *Nat. Commun.* **15**, 10731 (2024).

75. L. Chee, G. Valle, M. Marazzi, G. Preatoni, F. L. Haufe, M. Xiloyannis, R. Riener, S. Raspopovic, Optimally-calibrated non-invasive feedback improves amputees' metabolic consumption, balance and walking confidence. *J. Neural Eng.* **19**, 046049 (2022).

76. L. Chee, G. Valle, G. Preatoni, C. Basla, M. Marazzi, S. Raspopovic, Cognitive benefits of using non-invasive compared to implantable neural feedback. *Sci. Rep.* **12**, 16696 (2022).

77. G. V. Aurucci, N. Gozzi, M. Wagner, G. Preatoni, N. Brunello, A. Cimolato, N. Secerovic, C. M. Zipser, S. Raspopovic, Targeted neural stimulation congruent with immersive reality decreases neuropathic pain. *Brain Stimul.* **18**, 1671–1674 (2025).

78. N. Smania, A. Picelli, M. Daniele, C. Geroin, P. Ianes, A. Waldner, M. Gandolfi, Rehabilitation procedures in the management of spasticity. *Eur. J. Phys. Rehabil. Med.* **46**, 423–438 (2010).

79. S. Mateo, P. Revol, M. Fourtassi, Y. Rossetti, C. Collet, G. Rode, Kinematic characteristics of tenodesis grasp in C6 quadriplegia. *Spinal Cord* **51**, 144–149 (2013).

80. D. Yarnitsky, Quantitative sensory testing. *Muscle Nerve* **20**, 198–204 (1997).

81. S. R. Soekadar, M. Witkowski, C. Gómez, E. Opisso, J. Medina, M. Cortese, M. Cempini, M. C. Carrozza, L. G. Cohen, N. Birbaumer, N. Vitiello, Hybrid EEG/EOG-based brain/neural hand exoskeleton restores fully independent daily living activities after quadriplegia. *Sci. Robot.* **1**, eaag3296 (2016).

82. C. Stein, C. G. Fritsch, C. Robinson, G. Sbruzzi, R. D. M. Plentz, Effects of electrical stimulation in spastic muscles after stroke. *Stroke* **46**, 2197–2205 (2015).

83. S. R. Thomaz, G. Cipriano Jr., M. F. Formiga, E. Fachin-Martins, G. F. B. Cipriano, W. R. Martins, L. P. Cahalin, Effect of electrical stimulation on muscle atrophy and spasticity in patients with spinal cord injury – a systematic review with meta-analysis. *Spinal Cord* **57**, 258–266 (2019).

84. S. Raspopovic, G. Valle, F. M. Petrini, Sensory feedback for limb prostheses in amputees. *Nat. Mater.* **20**, 925–939 (2021).

85. C. Basla, L. Chee, G. Valle, S. Raspopovic, A non-invasive wearable sensory leg neuroprosthesis: Mechanical, electrical and functional validation. *J. Neural Eng.* **19**, 016008 (2022).

86. X. L. Hu, K. Y. Tong, R. Li, M. Chen, J. J. Xue, S. K. Ho, P. N. Chen, "Post-stroke wrist rehabilitation assisted with an intention-driven functional electrical stimulation (FES)-robot system," in *2011 IEEE International Conference on Rehabilitation Robotics* (IEEE, 2011), pp. 1–6.

87. G. Aiello, G. Valle, S. Raspopovic, Recalibration of neuromodulation parameters in neural implants with adaptive Bayesian Optimization. *J. Neural Eng.* **20**, 026037 (2023).

88. Y. Hara, Brain plasticity and rehabilitation in stroke patients. *J. Nippon Med. Sch.* **82**, 4–13 (2015).

89. B. Ravneberg, Usability and abandonment of assistive technology. *J. Assist. Technol.* **6**, 259–269 (2012).

90. A. E. Pena, J. J. Abbas, R. Jung, Channel-hopping during surface electrical neurostimulation elicits selective, comfortable, distally referred sensations. *J. Neural Eng.* **18**, 055004 (2021).

91. T. du Plessis, K. Djouani, C. Oosthuizen, A review of active hand exoskeletons for rehabilitation and assistance. *Robotics* **10**, 40 (2021).

92. L. Borda, N. Gozzi, G. Preatoni, G. Valle, S. Raspopovic, Automated calibration of somatosensory stimulation using reinforcement learning. *J. NeuroEngineering Rehabil.* **20**, 131 (2023).

93. R. Alicea, M. Xiloyannis, D. Chiaradia, M. Barsotti, A. Frisoli, L. Masia, A soft, synergy-based robotic glove for grasping assistance. *Wearable Technol.* **2**, e4 (2021).

94. R. Hennig, J. Gantenbein, J. Dittli, H. Chen, S. P. Lacour, O. Lambercy, R. Gassert, “Development and evaluation of a sensor glove to detect grasp intention for a wearable robotic hand exoskeleton,” in *2020 8th IEEE RAS/EMBS International Conference for Biomedical Robotics and Biomechatronics* (*BioRob*) (IEEE, 2020), pp. 19–24.

95. J. J. Gerhardt, R. D. Rondinelli, Goniometric techniques for range-of-motion assessment. *Phys. Med. Rehabil. Clin. N. Am.* **12**, 507–528 (2001).

96. F. Clemente, M. D’Alonzo, M. Controzzi, B. B. Edin, C. Cipriani, Non-invasive, temporally discrete feedback of object contact and release improves grasp control of closed-loop myoelectric transradial prostheses. *IEEE Trans. Neural Syst. Rehabil. Eng.* **24**, 1314–1322 (2016).

97. J. Arata, K. Ohmoto, R. Gassert, O. Lambercy, H. Fujimoto, I. Wada, “A new hand exoskeleton device for rehabilitation using a three-layered sliding spring mechanism: 2013 IEEE International Conference on Robotics and Automation, ICRA 2013,” in *2013 IEEE International Conference on Robotics and Automation*, *ICRA 2013* (IEEE, 2013), pp. 3902–3907.

98. J. Dittli, J. T. Meyer, J. Gantenbein, T. Bützer, R. Ranzani, A. Linke, A. Curt, R. Gassert, O. Lambercy, Mixed methods usability evaluation of an assistive wearable robotic hand orthosis for people with spinal cord injury. *J. Neuroeng. Rehabil.* **20**, 162 (2023).

99. T. Bützer, J. Dittli, J. Lieber, H. J. A. van Hedel, A. Meyer-Heim, O. Lambercy, R. Gassert, “PEXO - A pediatric whole hand exoskeleton for grasping assistance in task-oriented training,” in *2019 IEEE 16th International Conference on Rehabilitation Robotics* (*ICORR*) (IEEE, 2019), pp. 108–114.

100. H. In, B. B. Kang, M. Sin, K.-J. Cho, Exo-Glove: A wearable robot for the hand with a soft tendon routing system. *IEEE Robot. Automat. Mag.* **22**, 97–105 (2015).

101. M. Sierotowicz, N. Lotti, L. Nell, F. Missiroli, R. Alicea, X. Zhang, M. Xiloyannis, R. Rupp, E. Papp, J. Krzywinski, C. Castellini, L. Masia, EMG-driven machine learning control of a soft glove for grasping assistance and rehabilitation. *IEEE Robot. Autom. Lett.* **7**, 1566–1573 (2022).

102. F. Missiroli, N. Lotti, E. Tricomi, C. Bokranz, R. Alicea, M. Xiloyannis, J. Krzywinski, S. Crea, N. Vitiello, L. Masia, Rigid, soft, passive, and active: A hybrid occupational exoskeleton for bimanual multijoint assistance. *IEEE Robotics Autom. Lett.* **7**, 2557–2564 (2022).
